# Molecular basis of coronary artery disease–malignancy comorbidity: inflammation, immunometabolism, thrombosis, and cardio-oncology translation

**DOI:** 10.3389/fimmu.2026.1903190

**Published:** 2026-08-12

**Authors:** Junlin Li, Yan Zhao, Ming Bai

**Affiliations:** Department of Cardiovascular Medicine, Lanzhou University First Clinical Medical College, Lanzhou, China

**Keywords:** cardio-oncology, clonal hematopoiesis, coronary artery disease, immunometabolism, malignancy

## Abstract

Coronary artery disease (CAD) and malignancy frequently coexist in aging populations and share multiple risk factors, including smoking, obesity, diabetes, dyslipidemia, chronic inflammation, and metabolic dysfunction. However, increasing evidence suggests that CAD–malignancy comorbidity cannot be fully explained by epidemiological coincidence alone, although its causal direction remains difficult to establish because of shared risk factors, surveillance bias, reverse causality, cancer stage, and treatment exposure. Instead, convergent inflammatory, metabolic, thrombotic, and immune mechanisms may contribute to the observed clinical overlap. This review summarizes recent evidence linking CAD and cancer through chronic systemic inflammation, clonal hematopoiesis, inflammasome activation, immunometabolic remodeling, endothelial dysfunction, platelet activation, neutrophil extracellular trap formation, coagulation, and immune checkpoint disruption. Particular attention is given to clonal hematopoiesis as a molecular bridge between malignant predisposition and atherosclerosis, macrophage and T-cell immunometabolism as shared immune programs, and thromboinflammation as a mechanism connecting cancer-associated thrombosis with coronary vascular events. We further discuss cardio-oncology translation, including immune checkpoint inhibitor-related cardiovascular toxicity, CHIP-guided anti-inflammatory prevention, antiplatelet and antithrombotic strategies, endothelial-targeted interventions, and immune checkpoint-aware cardiovascular monitoring. Understanding these shared pathways may help move cardio-oncology beyond the management of therapy-induced cardiotoxicity toward mechanism-guided prevention and treatment of inflammatory, thrombotic, and immune mechanisms that jointly promote CAD progression and cancer development.

## Highlights

- CAD–malignancy comorbidity is driven by shared inflammatory, immunometabolic, thrombotic, and immune checkpoint-related mechanisms.
- Clonal hematopoiesis links cancer predisposition to atherosclerosis through macrophage remodeling and inflammasome activation.
- Precision cardio-oncology may require CHIP profiling, inflammatory biomarkers, thrombosis assessment, plaque imaging, and immune checkpoint-aware monitoring.

## 1 Introduction

Coronary artery disease (CAD) and malignancy frequently coexist, particularly in aging populations, reflecting not only shared lifestyle and metabolic risk factors but also overlapping pathophysiological mechanisms (1–5). Traditional risk factors—including smoking, obesity, diabetes, chronic inflammation, dyslipidemia, immune senescence, and metabolic dysfunction—predispose individuals to both atherosclerotic cardiovascular disease and cancer. While these shared risk factors partially explain their co-occurrence, emerging evidence indicates that the association between CAD and malignancy is more than epidemiological coincidence, and may instead reflect convergent molecular programs that simultaneously predispose to vascular and oncologic disease (6–10).

Several clinical studies have substantiated this concept. For example, Sun et al. demonstrated that systemic inflammation significantly modifies the association between cancer and CAD severity, indicating that inflammatory status acts as a key mediator rather than a passive correlate (11). Similarly, Zhao et al. reported that in lung cancer patients, the severity of coronary artery disease was more strongly associated with malignancy under high systemic inflammatory conditions, highlighting the importance of context-dependent immune activation (12). Large cohort analyses have also shown that patients with stable CAD exhibit an increased risk of subsequent cancer incidence and cancer-related mortality, with elevated levels of inflammatory and metabolic biomarkers serving as predictive indicators (13). Li et al. further corroborated these findings, providing evidence that CAD and cancer share overlapping risk profiles across diverse populations, suggesting that common pathophysiological pathways, rather than chance, drive their co-occurrence (14). Together, these studies suggest that CAD–malignancy comorbidity is a clinically significant phenomenon, in which inflammation, immune dysregulation, and metabolic perturbations may act as key mechanistic links. This recognition has important implications for risk stratification, patient monitoring, and the development of integrated cardio-oncology strategies.

Based on clinical and mechanistic evidence, we propose that comorbidity between coronary artery disease (CAD) and malignancy arises from a dynamic, multilayered interplay rather than from shared risk factors alone. Persistent low-grade systemic inflammation drives endothelial dysfunction, leukocyte recruitment, and plaque instability in CAD, while simultaneously fostering tumor initiation, angiogenesis, immune evasion, and metastatic progression (15–19). Key inflammatory mediators such as IL-1β, IL-6, TNF-α, and chemokine gradients create a permissive microenvironment that bridges vascular and oncologic pathology. This inflammatory milieu is further shaped by immunometabolic reprogramming of innate and adaptive immune cells: macrophages and monocytes undergo glycolytic and lipid metabolic shifts, while T cells experience clonal expansion and checkpoint modulation. Such alterations contribute to chronic vascular inflammation and tumor-promoting immune suppression, with clonal hematopoiesis and inflammasome activation serving as central mechanistic drivers linking cardiovascular and cancer-associated immune remodeling.

**Figure 1:**
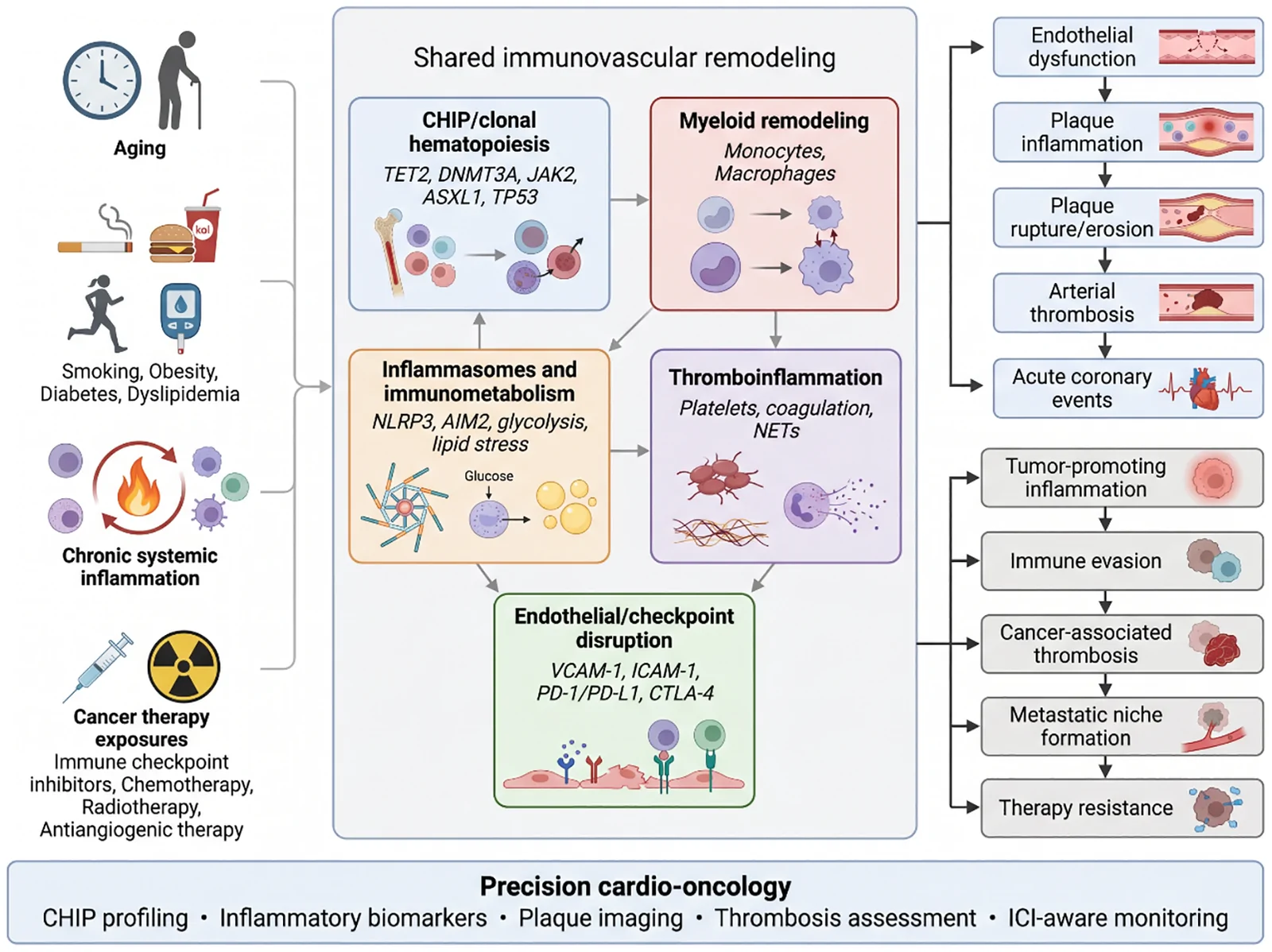
Central mechanistic model of CAD–malignancy comorbidity. Shared drivers, including aging, cardiometabolic risk factors, and cancer therapy, promote CHIP-driven myeloid remodeling. This hub amplifies inflammasome activation, immunometabolic stress, thromboinflammation, and endothelial/checkpoint disruption, thereby contributing to CAD progression, tumor-promoting inflammation, immune evasion, cancer-associated thrombosis, and metastasis.

Beyond inflammation and immunometabolism, thromboinflammatory processes and therapy-related vascular perturbations further integrate CAD and malignancy. Platelet hyperactivation, prothrombotic endothelial states, and neutrophil extracellular trap formation promote vascular occlusion in CAD, while simultaneously facilitating tumor cell survival, immune evasion, and metastatic dissemination. Moreover, anticancer treatments—including immune checkpoint inhibitors and chemotherapies—can exacerbate cardiovascular risk by inducing immune activation, endothelial injury, or off-target cardiotoxicity, potentially destabilizing pre-existing plaques or triggering myocardial inflammation. Collectively, these interconnected processes—encompassing inflammation, immunometabolism, thromboinflammation, and therapy-induced vascular stress—constitute a unified framework for understanding CAD–malignancy comorbidity and provide a mechanistic basis for integrated clinical management, early detection, and targeted intervention at the interface of cardiology and oncology. Importantly, the CAD–malignancy association should not be interpreted as evidence of direct causation. Several major confounders may contribute to their co-occurrence, including advanced age, smoking, obesity, diabetes, dyslipidemia, hypertension, chronic kidney disease, systemic inflammation, cancer stage, and anticancer treatment exposure. In addition, surveillance bias may increase cancer detection among patients with CAD who undergo frequent medical evaluation, whereas reverse causality may occur when occult malignancy promotes inflammation, hypercoagulability, cachexia, or vascular events before cancer diagnosis. Therefore, this review interprets CAD–malignancy comorbidity as a clinically observed overlap shaped by both shared risk factors and biologically plausible mechanisms, rather than as a simple one-directional causal relationship. **Figure 1** summarizes the central framework of CAD–malignancy comorbidity as a convergent immunovascular network rather than several isolated mechanisms. CHIP-driven myeloid remodeling is positioned as the key hub linking shared risk factors, inflammation, thrombosis, vascular injury, immune dysregulation, and cancer therapy-related stress.

## 2 Epidemiological and clinical evidence linking CAD and malignancy

### 2.1 CAD as a cancer-associated clinical phenotype

A growing body of clinical evidence indicates that coronary artery disease (CAD) and malignancy are epidemiologically and biologically interconnected. Patients with established CAD may exhibit an increased risk of subsequent cancer incidence and cancer-related mortality, whereas patients with cancer frequently develop cardiovascular complications, including ischemic heart disease, arterial thrombosis, and accelerated vascular dysfunction (20–24). This bidirectional relationship suggests that CAD should not be viewed solely as a competing comorbidity in oncology patients, but also as a potential marker of a systemic disease state characterized by chronic inflammation, metabolic dysregulation, vascular injury, and immune remodeling.

Campolo et al. provided important evidence supporting this concept by evaluating clinical and biological predictors of cancer incidence and mortality in patients with stable CAD (13). Their findings suggest that patients with CAD may represent a biologically vulnerable population in whom inflammatory and metabolic abnormalities contribute not only to cardiovascular events but also to cancer development and cancer-related death. This observation is important because it shifts the CAD–cancer relationship from a purely risk-factor-based association to a broader pathobiological framework. In this context, CAD may reflect long-standing exposure to systemic inflammatory and metabolic stressors that also favor oncogenesis. Similarly, Li et al. examined the interrelationship between coronary atherosclerotic disease and cancer and demonstrated that the two disease entities share overlapping clinical risk profiles (14). Age, smoking, diabetes, obesity, dyslipidemia, and chronic inflammatory burden are common determinants of both atherosclerosis and malignancy. However, these shared factors may not fully explain the observed association. Instead, they may interact with deeper biological processes, such as endothelial dysfunction, immune-cell activation, oxidative stress, and thromboinflammation, which create a permissive environment for both plaque progression and tumor initiation.

Therefore, CAD may be interpreted as a cancer-associated clinical phenotype in selected populations, particularly when accompanied by persistent inflammation or metabolic dysfunction. This does not imply that CAD directly causes cancer in all patients, but rather that CAD may serve as a visible vascular manifestation of systemic biological programs that also contribute to malignancy risk. Recognizing this possibility is clinically relevant because it supports more integrated screening, risk stratification, and long-term surveillance strategies in patients with either disease.

### 2.2 Cancer type-specific association with CAD

The relationship between CAD and malignancy is unlikely to be uniform across all tumor types. Different cancers vary substantially in their inflammatory phenotype, metabolic activity, thrombotic potential, treatment exposure, and association with traditional cardiovascular risk factors. Lung cancer is one of the most representative examples of CAD–malignancy overlap because it shares several major pathogenic drivers with coronary atherosclerosis, including cigarette smoking, chronic pulmonary and systemic inflammation, endothelial injury, oxidative stress, hypercoagulability, and immune activation (25–29). Zhao et al. showed that the anatomical severity of CAD was more strongly associated with lung cancer in patients with higher inflammatory status (12). This finding suggests that inflammation may act as a context-dependent amplifier of the CAD–cancer relationship. In patients with low inflammatory burden, shared risk factors such as age and smoking may explain much of the overlap. In contrast, in patients with high inflammatory burden, activated immune and vascular pathways may strengthen the biological connection between atherosclerosis and malignancy. Sun et al. further supported this concept by showing that inflammation modifies the association between cancer and CAD severity (11). This observation is particularly important because it implies that the CAD–malignancy relationship is not static. Rather, it may depend on the host inflammatory state, immune-cell composition, and vascular microenvironment. Inflammatory biomarkers may therefore help identify cancer patients who are more likely to harbor advanced coronary atherosclerosis, as well as CAD patients who may be at increased risk of malignancy.

Cancer type-specific analysis is also important from a mechanistic perspective. Lung cancer may be strongly linked to CAD through smoking-induced endothelial injury and chronic inflammation. Pancreatic and gastric cancers may be more closely associated with hypercoagulability, platelet activation, and cancer-associated thrombosis. Hematologic malignancies or premalignant hematopoietic clones may be connected to CAD through clonal hematopoiesis and inflammasome activation. These differences indicate that CAD–malignancy comorbidity should not be studied as a single homogeneous entity. Instead, future research should define tumor-specific cardiovascular phenotypes according to inflammatory status, thrombotic risk, immune profile, and treatment exposure.

### 2.3 From association to mechanism: confounding, causality, and biological plausibility

Before moving from epidemiological association to mechanistic interpretation, several methodological limitations must be considered. CAD and malignancy share many powerful upstream determinants, including aging, smoking, obesity, diabetes, dyslipidemia, hypertension, sedentary lifestyle, and chronic inflammatory burden (2, 30). These factors may confound observational associations and make it difficult to determine whether CAD promotes cancer, cancer accelerates CAD, or both conditions arise from shared systemic biology. Surveillance bias is also relevant, because patients with established CAD often undergo more frequent clinical contact, imaging, and laboratory testing, which may increase the likelihood of cancer detection (1, 3). Conversely, reverse causality should be considered because undiagnosed malignancy can induce inflammation, anemia, hypercoagulability, weight loss, endothelial injury, or thrombotic events before formal cancer diagnosis. Accordingly, the mechanisms discussed below should be viewed as biologically plausible and increasingly testable pathways that may explain part of the CAD–malignancy overlap, rather than as definitive proof of direct causation in all patients.

Although traditional risk factors such as aging, smoking, diabetes, obesity, hypertension, and dyslipidemia explain part of the clinical overlap between CAD and cancer, they do not fully account for the depth and complexity of CAD–malignancy comorbidity. The emerging challenge is to move beyond epidemiological association and identify the molecular mechanisms that simultaneously promote vascular disease and malignant progression.

Several candidate mechanisms are increasingly recognized. First, clonal hematopoiesis provides a compelling link between cancer predisposition and atherosclerosis. Age-associated hematopoietic clones carrying mutations in genes such as TET2, DNMT3A, JAK2, and ASXL1 can increase the risk of hematologic malignancy while also promoting vascular inflammation through altered monocyte and macrophage function (31–37). Second, macrophage inflammasome activation may serve as a central inflammatory mechanism connecting mutated hematopoietic clones, lipid accumulation, cytokine release, and plaque progression (38–42). Third, adaptive immune dysregulation, including T-cell clonal expansion, immune checkpoint imbalance, and loss of tolerance, may contribute to both tumor immune remodeling and atherosclerotic plaque inflammation. In addition, endothelial activation, platelet–tumor cell crosstalk, neutrophil extracellular trap formation, and coagulation cascade activation provide important thromboinflammatory links between cancer and CAD (43–47). In CAD, these processes contribute to plaque instability, arterial thrombosis, and acute coronary events. In cancer, they support tumor cell survival in circulation, metastatic seeding, immune evasion, and cancer-associated thrombosis. These shared mechanisms suggest that thrombosis is not merely a downstream complication but an active biological interface between cardiovascular and oncologic disease.

**Figure 2:**
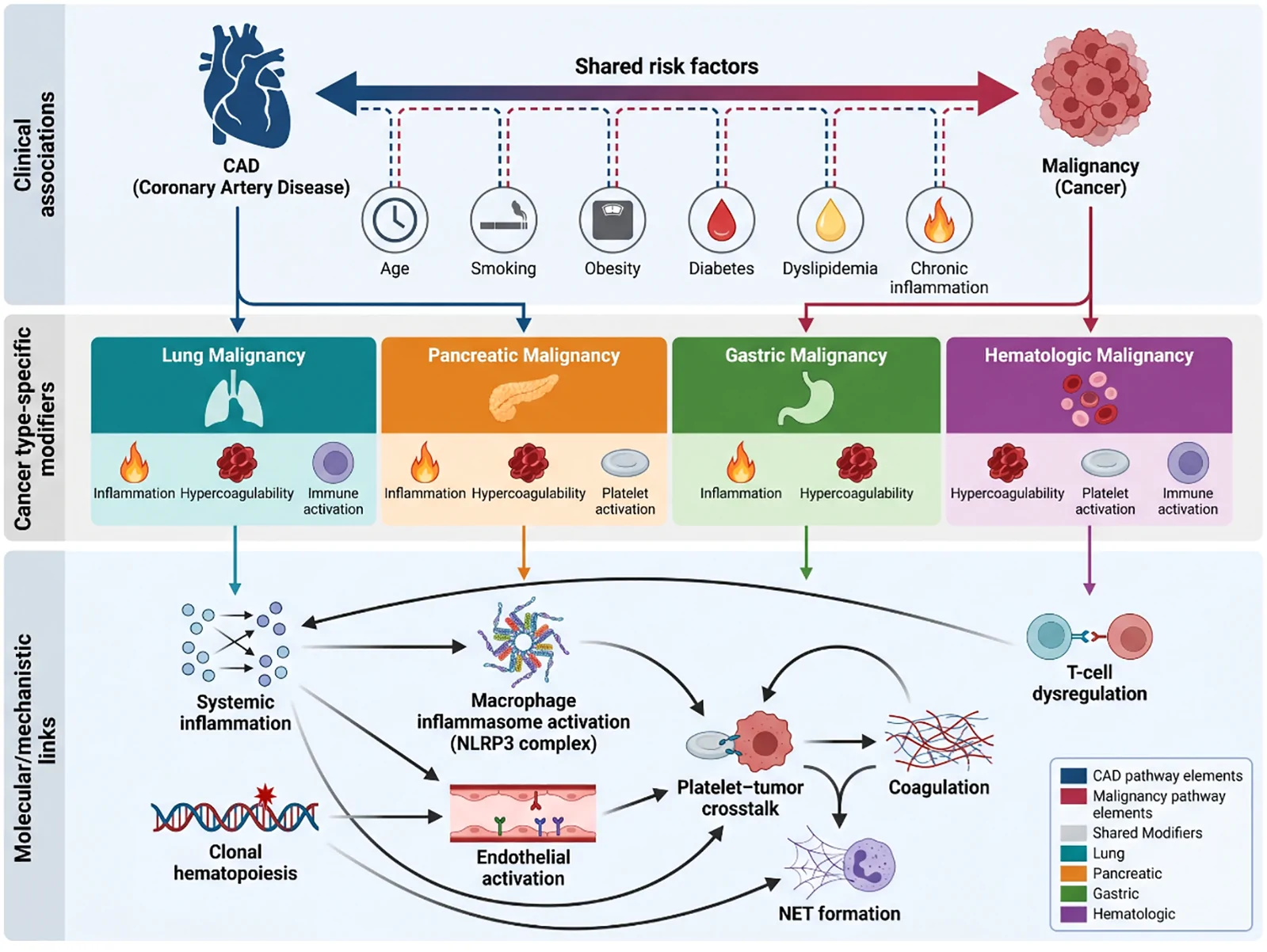
Mechanistic overview of CAD–malignancy comorbidity. Shared clinical risk factors (age, smoking, obesity, diabetes, dyslipidemia, chronic inflammation) converge with tumor type-specific modifiers (inflammation, hypercoagulability, platelet activation) and molecular pathways (clonal hematopoiesis, inflammasome activation, immune dysregulation, endothelial dysfunction, NETs, coagulation) to drive bidirectional cardiovascular and cancer pathology. Arrows indicate key interactions and amplification loops connecting vascular and oncologic disease processes.

Thus, the CAD–malignancy relationship should be understood as a continuum that progresses from shared clinical risk factors to shared molecular drivers. Epidemiological studies establish the clinical relevance of this comorbidity, whereas mechanistic studies explain how inflammation, immunometabolism, clonal hematopoiesis, endothelial dysfunction, platelet activation, and coagulation jointly shape disease progression. This mechanistic transition provides the foundation for the following sections of this review, which will examine chronic inflammation, clonal hematopoiesis, immunometabolic remodeling, thrombosis, and cardio-oncology translation as interconnected layers of CAD–malignancy comorbidity. To summarize the current clinical evidence supporting the association between CAD and malignancy, we organized representative epidemiological and cohort studies according to disease context, inflammatory status, and major clinical findings (**Table 1**). **Figure 2** illustrates how CAD and malignancy are linked through overlapping clinical, immune, and thromboinflammatory mechanisms. It highlights both shared systemic risk factors and molecular pathways that jointly contribute to disease progression in heart and tumor tissues.

**Table 1:** Core clinical and epidemiologic evidence linking CAD and malignancy.

First author	Year	Study type	Key findings	CAD/cancer focus	PMID
Sun M	2022	Cohort	Systemic inflammation modifies association between CAD severity and cancer risk	General CAD–Cancer association	38267838
Zhao YW (12)	2022	Observational	CAD severity strongly associated with lung cancer under high inflammatory burden	Lung cancer	36339468
Campolo J (13)	2023	Cohort	Stable CAD patients exhibit increased cancer incidence and cancer-related mortality	General CAD–Cancer association	37446269
Li J (14)	2022	Multi-cohort	Bidirectional CAD–cancer association; shared risk factors include age, obesity, diabetes, inflammation	General CAD–Cancer association	35463783
Díez-Díez M	2023	Cohort/Mechanistic	CHIP associated with atherosclerosis progression; upstream driver	Hematologic malignancy & CAD	39215150

## 3 Chronic inflammation as a shared driver of CAD and malignancy

### 3.1 Systemic inflammation as a biological bridge

Chronic low-grade inflammation represents one of the most important biological links between coronary artery disease (CAD) and malignancy. In CAD, persistent inflammatory activation promotes endothelial dysfunction, leukocyte adhesion, monocyte recruitment, macrophage foam-cell formation, vascular smooth muscle cell remodeling, plaque growth, and plaque destabilization (48–53). In malignancy, inflammatory signaling contributes to tumor initiation, angiogenesis, immune escape, extracellular matrix remodeling, metastatic dissemination, and therapy resistance. Therefore, inflammation provides a shared pathogenic language through which vascular and oncologic diseases may influence each other.

The inflammatory connection between CAD and cancer is supported by clinical evidence showing that the association between these diseases is strengthened in patients with higher inflammatory burden. Sun et al. demonstrated that systemic inflammation modifies the relationship between cancer and the anatomical severity of CAD (11). This finding is important because it suggests that inflammation is not simply a biomarker reflecting disease burden, but may actively shape the CAD–malignancy interface. In this context, patients with cancer and high inflammatory status may be more likely to harbor advanced coronary atherosclerosis, whereas patients with CAD and persistent inflammation may represent a population with increased vulnerability to malignancy. A similar pattern was observed in lung cancer. Zhao et al. reported that the association between CAD severity and lung cancer was more evident under high inflammatory conditions (12). Lung cancer is particularly relevant to this discussion because it shares major inflammatory and vascular risk factors with CAD, including cigarette smoking, chronic oxidative stress, pulmonary inflammation, endothelial injury, and systemic immune activation. These findings indicate that the CAD–cancer relationship is context-dependent and may be amplified by inflammatory status, cancer type, and host immune background. Mechanistically, systemic inflammation can promote CAD–malignancy comorbidity through several interconnected pathways. Pro-inflammatory cytokines such as IL-1β, IL-6, TNF-α, and chemokines can activate endothelial cells and increase the expression of adhesion molecules, thereby facilitating leukocyte recruitment into the vascular wall (54–57). In parallel, these mediators can promote tumor-cell proliferation, angiogenesis, epithelial–mesenchymal transition, and immune suppression within the tumor microenvironment. Chronic inflammation also induces oxidative stress and DNA damage, which contribute to both vascular injury and genomic instability. Moreover, inflammatory signaling can enhance tissue factor expression, platelet activation, neutrophil extracellular trap formation, and coagulation cascade activation, creating a prothrombotic state that is relevant to both acute coronary events and cancer-associated thrombosis.

Thus, systemic inflammation should be regarded as a central organizing mechanism of CAD–malignancy comorbidity. It connects traditional cardiovascular risk factors, tumor-promoting microenvironments, immune-cell remodeling, and thrombotic complications into a unified biological framework. Importantly, this inflammatory bridge also provides a rationale for identifying high-risk patients through inflammatory biomarkers and for testing anti-inflammatory strategies in selected cardio-oncology populations.

### 3.2 Inflammasomes in the CAD–cancer interface

Inflammasomes are cytosolic multiprotein complexes that detect cellular stress, microbial products, danger-associated molecular patterns, and endogenous damage signals. Once activated, inflammasomes promote caspase-1 activation, maturation of IL-1β and IL-18, and inflammatory cell death. Among them, AIM2 and NLRP3 inflammasomes are particularly relevant to the CAD–cancer interface because they connect genomic instability, metabolic stress, innate immune activation, cytokine release, and vascular inflammation (58–60). In atherosclerosis, inflammasome activation contributes to macrophage inflammatory responses, endothelial dysfunction, plaque progression, and plaque instability. In cancer, inflammasome signaling can have context-dependent effects. It may promote antitumor immunity in some settings, but chronic inflammasome activation often supports tumor-promoting inflammation, angiogenesis, immune suppression, and metastatic niche formation. This dual role makes inflammasomes highly relevant to CAD–malignancy comorbidity, where persistent innate immune activation may simultaneously aggravate vascular disease and create a cancer-permissive inflammatory environment.

Clonal hematopoiesis provides one of the most compelling mechanistic contexts in which inflammasomes link malignancy risk with atherosclerosis. In clonal hematopoiesis, age-associated hematopoietic stem and progenitor cell clones acquire mutations in genes such as JAK2, TET2, DNMT3A, and ASXL1 (61–63). These mutations increase the risk of hematologic malignancy and also reprogram myeloid cells toward heightened inflammatory activity. As a result, clonal hematopoiesis represents a biological bridge between cancer predisposition and vascular inflammation.

Fidler et al. provided strong mechanistic evidence for this concept by showing that JAK2V617F clonal hematopoiesis aggravates atherosclerosis through macrophage proliferation, glycolytic reprogramming, DNA replication stress, and AIM2 inflammasome activation (64). This study is particularly important because it links a cancer-associated hematopoietic mutation to vascular disease through a defined innate immune pathway. Rather than acting merely as a hematologic risk marker, mutant hematopoietic clones can generate inflammatory macrophage populations that directly accelerate atherosclerotic disease. In parallel, Yalcinkaya et al. demonstrated that TET2 clonal hematopoiesis promotes atherosclerosis through JNK1–BRCC3-mediated NLRP3 deubiquitylation and inflammasome activation (65). This study expands the concept beyond AIM2 and highlights NLRP3 as another key inflammasome pathway connecting clonal hematopoiesis, lipid stress, and vascular inflammation. The finding that BRCC3-mediated deubiquitylation stabilizes or activates NLRP3 signaling suggests that post-translational regulation of inflammasome components may be an important layer of CAD–malignancy biology.

Together, these studies support a model in which cancer-prone hematopoietic clones generate inflammatory myeloid cells that are metabolically rewired and primed for inflammasome activation. Once recruited to vascular lesions, these cells produce IL-1β, IL-18, and other inflammatory mediators that amplify endothelial activation, leukocyte recruitment, and plaque progression. At the same time, systemic inflammasome-driven inflammation may support tumor-promoting immune remodeling and cancer-related thromboinflammation. Therefore, AIM2 and NLRP3 inflammasomes represent molecular nodes where genomic instability, immunometabolism, vascular inflammation, and malignancy risk converge.

### 3.3 Anti-inflammatory intervention as translational opportunity

Because inflammation is a central mechanism linking CAD and malignancy, it also represents a potential therapeutic vulnerability. Anti-inflammatory strategies may be particularly valuable in patients with persistent systemic inflammation, clonal hematopoiesis, cancer-associated inflammatory syndromes, or established cardiovascular disease (66–70). Rather than applying anti-inflammatory therapy broadly to all patients, future cardio-oncology approaches may need to identify specific inflammatory endotypes that are most likely to benefit. The translational relevance of this concept is illustrated by Zuriaga et al. who showed that colchicine prevents accelerated atherosclerosis in TET2-mutant clonal hematopoiesis (71). This study provides an important proof of principle: genetically defined inflammatory cardiovascular risk can potentially be mitigated by targeted anti-inflammatory intervention. For CAD–malignancy comorbidity, this finding has major implications. Patients carrying TET2-mutant clonal hematopoiesis may be at increased risk for both hematologic malignancy and inflammatory vascular disease. In such patients, anti-inflammatory therapy could theoretically reduce cardiovascular risk while also modulating systemic inflammatory tone.

Colchicine is especially relevant because it targets inflammatory processes involved in neutrophil activation, inflammasome signaling, and cytokine release. In the setting of clonal hematopoiesis, where mutant myeloid cells are primed toward excessive inflammatory responses, colchicine may help restrain the downstream vascular consequences of these clones. This raises the possibility that CHIP screening, inflammatory biomarker profiling, and vascular imaging could be integrated into future precision cardio-oncology strategies.

However, several important challenges remain. First, inflammation is not uniformly harmful in cancer. Some inflammatory pathways are required for effective antitumor immunity, antigen presentation, and response to immunotherapy. Broad suppression of inflammation may therefore have different effects depending on cancer type, immune contexture, and treatment exposure (72–76). Second, patients with cancer often have competing risks, including infection, thrombocytopenia, bleeding, drug–drug interactions, and impaired organ function, which may complicate anti-inflammatory treatment. Third, the optimal biomarkers for selecting patients remain unclear. Candidate markers may include high-sensitivity C-reactive protein, IL-6, IL-1β-related signatures, neutrophil-to-lymphocyte ratio, circulating monocyte phenotypes, and CHIP mutation status.

Despite these uncertainties, the anti-inflammatory approach represents one of the most promising translational directions in CAD–malignancy comorbidity. The key goal is not nonspecific suppression of inflammation, but mechanism-guided modulation of pathological inflammatory circuits. In the future, patients with cancer and CAD may be stratified according to inflammatory burden, clonal hematopoiesis genotype, plaque activity, thrombotic risk, and cancer therapy exposure. Such precision strategies could help identify individuals who may benefit from colchicine, inflammasome pathway inhibition, IL-1β/IL-6 axis modulation, or other targeted anti-inflammatory interventions.

**Figure 3:**
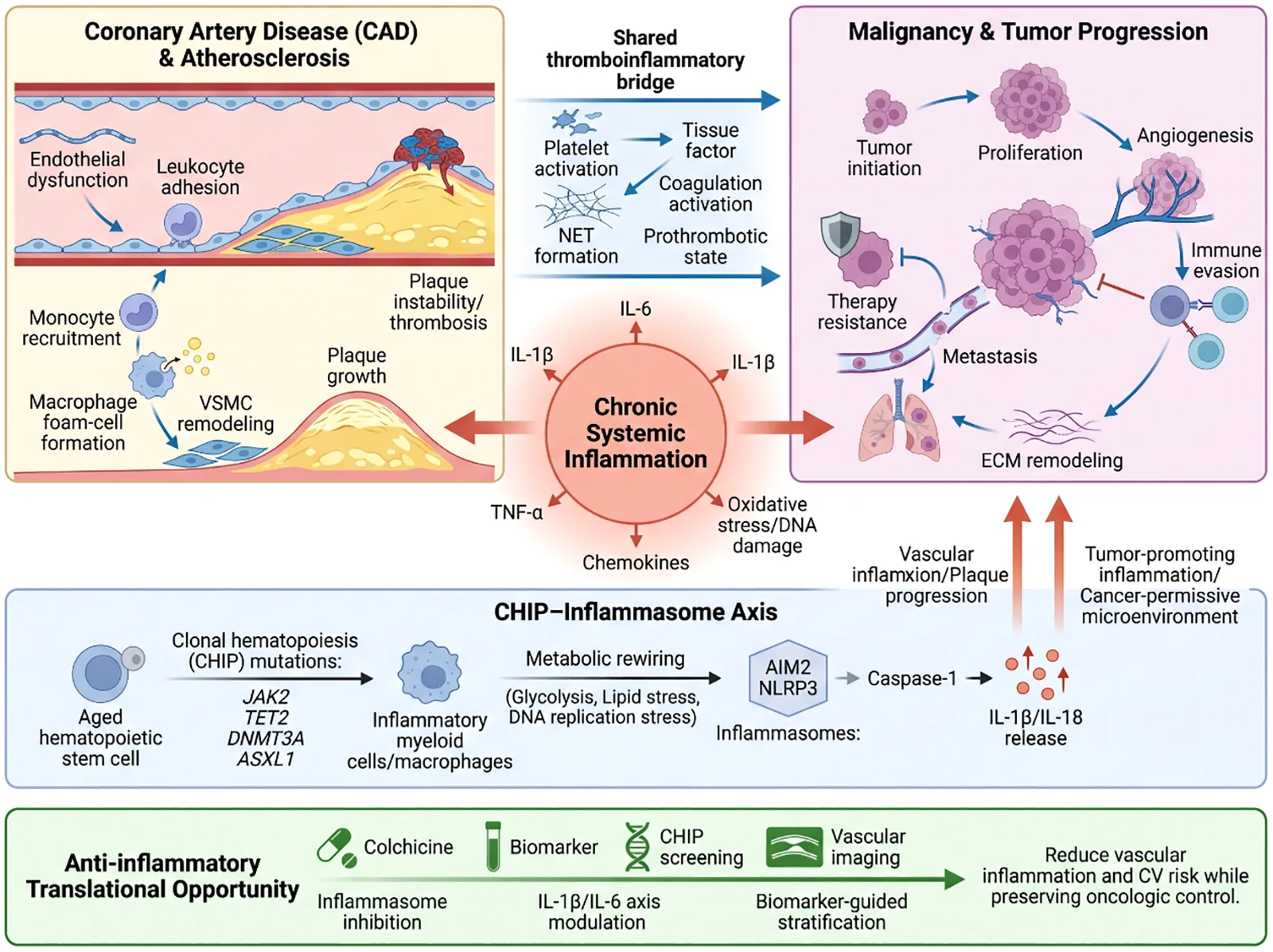
Chronic inflammation as a shared driver of CAD–malignancy comorbidity. Systemic inflammatory mediators promote endothelial dysfunction, leukocyte recruitment, plaque progression, tumor growth, angiogenesis, immune evasion, and thromboinflammation. Clonal hematopoiesis-associated myeloid remodeling activates AIM2 and NLRP3 inflammasomes, amplifying IL-1β/IL-18 release and vascular inflammation. Anti-inflammatory strategies, including colchicine and inflammasome pathway modulation, may provide translational opportunities for selected high-risk patients.

In summary, chronic inflammation is both a mechanistic driver and a therapeutic entry point in CAD–malignancy comorbidity. Clinical studies demonstrate that inflammatory burden modifies the CAD–cancer relationship, while mechanistic studies reveal that clonal hematopoiesis-associated AIM2 and NLRP3 inflammasome activation can accelerate atherosclerosis. These insights provide a strong foundation for developing inflammation-guided cardio-oncology strategies aimed at reducing cardiovascular risk without compromising oncologic control. **Figure 3** illustrates how chronic systemic inflammation links coronary artery disease and malignancy through shared vascular, immune, thrombotic, and tumor-promoting pathways. It also highlights CHIP-associated inflammasome activation as a mechanistic amplifier and anti-inflammatory intervention as a potential cardio-oncology strategy.

## 4 Clonal hematopoiesis: a molecular bridge between cancer predisposition and atherosclerosis

### 4.1 CHIP as a common origin of malignant and cardiovascular risk

Clonal hematopoiesis of indeterminate potential (CHIP) has emerged as one of the most compelling molecular links between malignancy and coronary artery disease (CAD) (77–81). CHIP refers to the age-associated expansion of hematopoietic stem and progenitor cell clones that carry somatic mutations, most commonly in genes such as TET2, DNMT3A, JAK2, and ASXL1, in individuals without overt hematologic malignancy. Although initially recognized as a premalignant condition that increases the risk of myeloid neoplasms, CHIP is now understood as a systemic inflammatory state that also contributes to cardiovascular disease.

The relevance of CHIP to CAD–malignancy comorbidity lies in its dual biological identity. On one hand, CHIP reflects an early stage of hematologic clonal evolution and therefore indicates increased malignant potential. On the other hand, CHIP-mutant immune cells, particularly monocytes and macrophages, can acquire pro-inflammatory, metabolically rewired, and plaque-promoting phenotypes. Thus, CHIP provides a shared cellular origin from which cancer predisposition and vascular inflammation may diverge but remain mechanistically connected. Díez-Díez et al. provided important clinical evidence supporting CHIP as an upstream driver of atherosclerosis development (82). In their longitudinal cohort, clonal hematopoiesis was associated with subsequent atherosclerosis progression, supporting the concept that CHIP is not merely a consequence of vascular disease or aging, but may actively promote the development of atherosclerotic lesions. Nevertheless, the term “unidirectional” should be interpreted within the specific cohort design and follow-up window. A highly hypercholesterolemic or chronically inflamed vascular milieu may, in turn, increase hematopoietic stem and progenitor cell proliferation, thereby facilitating clonal selection and expansion. Thus, CHIP and atherosclerosis may form a context-dependent feed-forward loop in which mutant myeloid cells promote plaque inflammation, while inflammatory or lipid-rich vascular environments further shape hematopoietic dynamics (83).

Mechanistic studies further support this model. Fidler et al. showed that JAK2V617F-driven clonal hematopoiesis accelerates atherosclerosis through macrophage proliferation, glycolytic reprogramming, DNA replication stress, and AIM2 inflammasome activation (64). This study is particularly important because JAK2V617F is a classical mutation associated with myeloproliferative neoplasms, yet it also drives vascular inflammation. Similarly, Yalcinkaya et al. demonstrated that TET2-mutant clonal hematopoiesis promotes atherosclerosis through JNK1–BRCC3-mediated NLRP3 deubiquitylation and inflammasome activation (65). Together, these studies indicate that different CHIP genotypes may converge on inflammatory myeloid pathways that accelerate atherosclerosis.

Therefore, CHIP should be viewed not only as a hematologic risk factor, but also as a molecular amplifier of CAD–malignancy comorbidity. It links aging, somatic mutation, inflammatory myelopoiesis, plaque progression, and malignant predisposition into a unified disease framework.

### 4.2 CHIP-driven immune-cell remodeling

A key mechanism through which CHIP promotes cardiovascular disease is immune-cell remodeling. CHIP mutations can alter the transcriptional, inflammatory, and metabolic states of hematopoietic-derived immune cells. Because monocytes, macrophages, neutrophils, dendritic cells, and T cells all arise from hematopoietic progenitors, clonal mutations can reshape systemic immunity long before overt hematologic malignancy develops. Myeloid cells are especially important in this process. CHIP-mutant monocytes and macrophages often display exaggerated inflammatory responses, increased cytokine production, altered lipid handling, and enhanced inflammasome activation (84–88). In the vascular wall, these cells can promote endothelial activation, foam-cell formation, necrotic core expansion, and plaque instability. In cancer, similar inflammatory myeloid programs may contribute to tumor-promoting inflammation, immune suppression, angiogenesis, and metastatic niche formation. Thus, CHIP-driven myeloid remodeling provides a shared immune mechanism that may influence both CAD progression and malignancy biology.

CHIP may also influence adaptive immunity. Although the effects of CHIP on T cells are less fully defined than those on myeloid cells, emerging evidence suggests that clonal hematopoiesis can alter immune-cell composition and inflammatory tone across multiple lineages. Abplanalp et al. demonstrated that DNMT3A mutations reshape immune cells in patients with heart failure, leading to altered monocyte and T-cell inflammatory profiles (89). Although this study focused on heart failure rather than CAD, it provides important evidence that CHIP-associated mutations can systemically reprogram immune-cell states. This concept is highly relevant to CAD–malignancy comorbidity because systemic immune remodeling may simultaneously influence vascular inflammation, tumor immune surveillance, and cancer-related immune escape. From a broader perspective, CHIP-driven immune remodeling may help explain why some patients develop both cardiovascular disease and malignancy despite similar traditional risk factors. In these individuals, the immune system itself may become a disease-promoting compartment. Mutated hematopoietic clones generate inflammatory immune cells that circulate throughout the body, infiltrate vascular lesions, interact with tumor microenvironments, and amplify tissue injury. Therefore, CHIP transforms hematopoiesis into a systemic source of inflammatory risk.

### 4.3 CHIP, immunometabolism, and inflammasome activation

Immunometabolic reprogramming is central to the pathological consequences of CHIP. Immune-cell activation requires metabolic adaptation, and CHIP-mutant cells appear to exploit this relationship to sustain chronic inflammation. In atherosclerosis, macrophages must adapt to lipid-rich, hypoxic, and inflammatory plaque microenvironments (90–93). In cancer, immune cells are exposed to nutrient deprivation, lactate accumulation, hypoxia, and tumor-derived metabolic signals. CHIP may predispose immune cells to respond to these environments in a more inflammatory and disease-promoting manner.

Fidler et al. provided a clear example of CHIP-associated immunometabolic remodeling (64). In JAK2V617F clonal hematopoiesis, macrophages exhibited increased proliferation and glycolytic activity. This metabolic shift was associated with DNA replication stress and activation of the AIM2 inflammasome. AIM2 senses cytosolic DNA and can trigger caspase-1 activation and IL-1β/IL-18 maturation. In this setting, a cancer-associated hematopoietic mutation creates metabolically activated macrophages that are prone to inflammasome signaling and capable of accelerating atherosclerosis. Yalcinkaya et al. demonstrated another important mechanism involving TET2 deficiency (65). In the presence of hypercholesterolemia, TET2-mutant macrophages showed increased NLRP3 inflammasome activation through JNK1–BRCC3-mediated NLRP3 deubiquitylation (94, 95). This finding highlights several important concepts. First, CHIP mutations do not act in isolation; their pathological effects are shaped by metabolic and environmental stressors such as hypercholesterolemia. Second, post-translational modification of inflammasome components can determine inflammatory output. Third, lipid stress and clonal hematopoiesis converge on innate immune activation, providing a direct mechanistic connection between dyslipidemia, CAD, and malignant predisposition.

Together, these studies suggest that CHIP-mutant macrophages function as immunometabolic amplifiers. They sense lipid accumulation, DNA stress, oxidative injury, and inflammatory cues, and respond by activating AIM2 or NLRP3 inflammasomes. The resulting production of IL-1β, IL-18, and other inflammatory mediators promotes endothelial activation, leukocyte recruitment, plaque growth, and plaque instability. At the systemic level, these cytokines may also contribute to cancer-promoting inflammation and suppression of effective antitumor immunity. This CHIP–immunometabolism–inflammasome axis is especially relevant to cardio-oncology. Cancer patients and survivors often have additional inflammatory and metabolic stressors, including chemotherapy-induced tissue injury, radiation exposure, immune checkpoint blockade, cachexia, obesity, diabetes, and dyslipidemia. In the presence of CHIP, these stressors may further amplify myeloid inflammation and vascular risk. Therefore, CHIP status may help explain heterogeneity in cardiovascular outcomes among cancer patients exposed to similar therapies.

### 4.4 CHIP-guided prevention in cardio-oncology

The recognition of CHIP as a shared driver of malignancy risk and cardiovascular inflammation opens new opportunities for precision cardio-oncology. Instead of evaluating cardiovascular risk using only traditional factors such as age, hypertension, smoking, diabetes, and lipid levels, future strategies may incorporate CHIP genotyping, inflammatory biomarkers, plaque imaging, and cancer therapy exposure. This approach may be particularly useful for cancer survivors, elderly patients, patients receiving immune-based therapies, and individuals with unexplained inflammatory cardiovascular risk. Zuriaga et al. provided a major proof-of-concept for genotype-informed prevention (71). Their study showed that colchicine prevents accelerated atherosclerosis in TET2-mutant clonal hematopoiesis. This finding is highly relevant because it demonstrates that CHIP-associated cardiovascular risk may be modifiable. Rather than treating CHIP as a fixed genetic risk marker, clinicians may eventually target the inflammatory pathways activated by specific CHIP genotypes.

In a cardio-oncology context, CHIP-guided prevention could have several potential applications. First, patients with known CHIP mutations may undergo more intensive cardiovascular surveillance, including assessment of coronary calcification, plaque burden, vascular inflammation, and circulating inflammatory biomarkers. Second, patients with TET2- or JAK2-mutant CHIP may be considered for anti-inflammatory or antithrombotic strategies if supported by future clinical trials (96–100). Third, CHIP status may help identify patients at higher risk of cardiovascular complications during cancer therapy, particularly when treatment induces systemic inflammation, endothelial injury, or immune activation. However, several challenges must be addressed before CHIP-guided prevention can be widely implemented. The clinical significance of CHIP depends on mutation type, variant allele fraction, co-mutations, age, comorbidities, and inflammatory context. Not all CHIP mutations carry the same cardiovascular or malignant risk. For example, JAK2 mutations may be particularly associated with thrombosis and myeloproliferative biology, whereas TET2 and DNMT3A mutations may have distinct inflammatory consequences. Therefore, CHIP should not be treated as a uniform entity. Genotype-specific risk models will be needed.

Another challenge is therapeutic balance. Anti-inflammatory strategies may reduce vascular risk, but excessive immune suppression may be undesirable in patients with active malignancy or those receiving immunotherapy. Similarly, antithrombotic therapy may reduce cardiovascular and cancer-associated thrombotic events but increase bleeding risk, especially in patients with thrombocytopenia, gastrointestinal tumors, or invasive procedures. Therefore, CHIP-guided cardio-oncology must integrate genetic, inflammatory, thrombotic, oncologic, and treatment-related variables. Despite these limitations, CHIP represents one of the most promising molecular entry points for understanding and managing CAD–malignancy comorbidity. It provides a biologically plausible explanation for why aging, cancer predisposition, vascular inflammation, and immune dysregulation often coexist. More importantly, it suggests that selected patients may benefit from mechanism-guided interventions aimed at mutant clone-driven inflammation.

In summary, clonal hematopoiesis is a central molecular bridge between malignancy and CAD. CHIP-mutant hematopoietic clones can increase malignant potential while simultaneously generating inflammatory immune cells that accelerate atherosclerosis. Through myeloid remodeling, immunometabolic reprogramming, AIM2 and NLRP3 inflammasome activation, and cytokine-driven vascular inflammation, CHIP provides a mechanistic foundation for CAD–malignancy comorbidity. Future cardio-oncology strategies may increasingly use CHIP status to guide cardiovascular surveillance, anti-inflammatory prevention, and individualized risk management.

### 4.5 Mutation-specific CHIP programs in CAD–malignancy comorbidity

**Figure 4:**
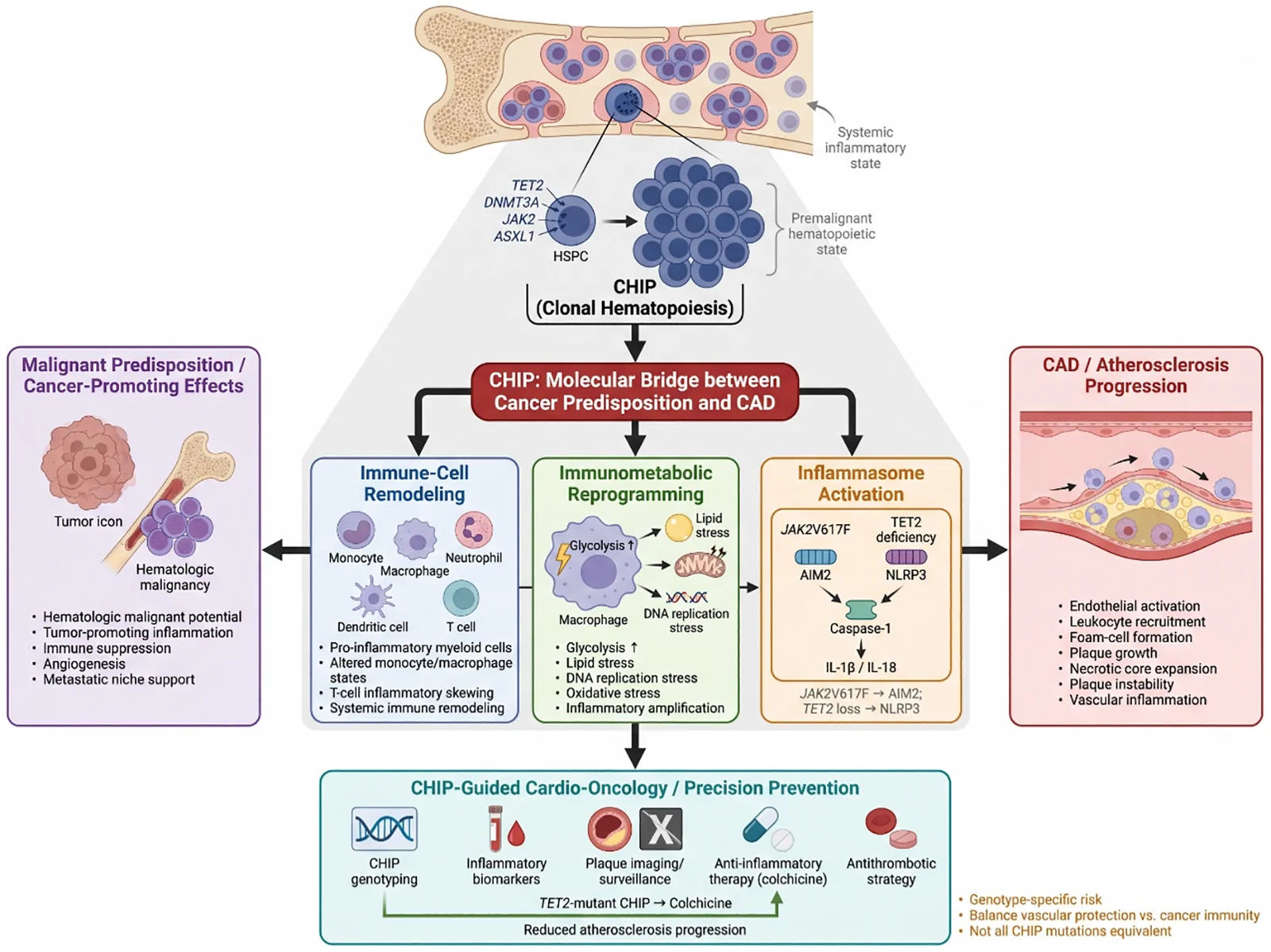
Clonal hematopoiesis (CHIP) links cancer predisposition and coronary artery disease (CAD) through immune-cell remodeling, immunometabolic reprogramming, and inflammasome activation. CHIP-mutant myeloid cells activate AIM2 and NLRP3 inflammasomes, producing IL-1β and IL-18, which amplify vascular inflammation, plaque progression, and tumor-promoting inflammation. CHIP-informed anti-inflammatory strategies, such as colchicine and biomarker-guided monitoring, represent translational opportunities to mitigate cardiovascular risk while preserving oncologic control.

Importantly, CHIP should not be treated as a biologically uniform entity. Different driver mutations may generate distinct inflammatory, thrombotic, metabolic, and malignant-risk phenotypes. TET2-mutant CHIP is most consistently linked to myeloid inflammatory remodeling, IL-1β-related signaling, and NLRP3 inflammasome activation, making it particularly relevant to inflammation-driven atherosclerosis (101, 102). DNMT3A-mutant CHIP is common in aging populations and may reshape immune-cell differentiation, inflammatory tone, and adverse cardiovascular remodeling, although its mechanistic connection to CAD appears less specific than that of TET2. By contrast, JAK2V617F-mutant CHIP is more closely associated with myeloproliferative biology, platelet–leukocyte activation, thrombosis, and heightened arterial or venous thrombotic risk (103, 104). ASXL1-mutant CHIP may contribute to inflammatory hematopoietic remodeling and adverse cardiovascular outcomes, but its CAD-specific mechanisms remain less well defined. TP53-mutant CHIP is especially relevant in cardio-oncology because it may expand after cytotoxic therapy or radiation and has been linked to therapy-related hematologic malignancy risk and cancer-treatment-associated cardiovascular vulnerability (105). Therefore, mutation-specific interpretation of CHIP may improve risk stratification and help distinguish inflammatory, thrombotic, and therapy-related CHIP endotypes in patients with CAD–malignancy comorbidity. To highlight this genotype-specific heterogeneity, **Table 2** summarizes the major CHIP mutations relevant to CAD–malignancy comorbidity and their potential mechanistic and translational implications. **Figure 4** illustrates how age-associated somatic mutations drive CHIP, generating inflammatory, metabolically reprogrammed myeloid cells that activate AIM2/NLRP3 inflammasomes. These processes concurrently promote atherosclerosis progression and malignancy risk, highlighting CHIP as a mechanistic bridge and a potential target for precision cardio-oncology interventions.

**Table 2:** Mutation-specific CHIP programs relevant to CAD–malignancy comorbidity.

CHIP mutation	Dominant biological phenotype	CAD-relevant mechanism	Malignancy/cardio-oncology relevance	Potential translational implication
TET2	Myeloid inflammatory remodeling; inflammasome activation	Promotes macrophage inflammatory activation, IL-1β/IL-6 signaling, NLRP3 inflammasome activation, endothelial inflammation, and plaque progression	Associated with myeloid neoplasm risk and systemic inflammatory tone; may amplify cancer-associated inflammatory states	Candidate for inflammation-guided surveillance; potential relevance to colchicine, IL-1β/NLRP3-axis modulation
DNMT3A	Age-related clonal expansion; altered immune-cell differentiation	May reshape monocyte and T-cell inflammatory profiles and contribute to adverse cardiovascular remodeling; CAD-specific mechanisms are less defined than TET2	Common CHIP driver in aging and cancer survivors; may influence immune fitness and hematologic risk	Requires integration with VAF, co-mutations, inflammatory biomarkers, and treatment exposure
JAK2V617F	Myeloproliferative and prothrombotic biology	Promotes platelet–leukocyte activation, thromboinflammation, endothelial activation, and arterial/venous thrombosis; may accelerate atherosclerosis through AIM2-related macrophage programs	Strongly linked to myeloproliferative neoplasms and thrombotic complications	May justify closer thrombosis assessment and individualized antiplatelet/antithrombotic consideration
ASXL1	Epigenetic dysregulation; inflammatory hematopoietic remodeling	May contribute to systemic inflammation and adverse cardiovascular outcomes, although CAD-specific mechanisms remain incompletely characterized	Associated with myeloid neoplasm risk and high-risk clonal evolution	Should be interpreted cautiously with age, VAF, co-mutations, and comorbidity burden
TP53	DNA damage response alteration; therapy-selected clonal expansion	May contribute to cancer-therapy-associated cardiovascular vulnerability, particularly after cytotoxic therapy or radiation; direct CAD mechanisms remain emerging	Strong relevance to therapy-related clonal hematopoiesis, therapy-related myeloid neoplasms, and cancer survivorship	Important in cardio-oncology surveillance after chemotherapy/radiotherapy; avoid treating as equivalent to inflammatory TET2-type CHIP

## 5 Immunometabolism in CAD–malignancy comorbidity

### 5.1 Monocyte–macrophage metabolic reprogramming

Macrophages are central mediators of both atherosclerotic plaque progression and tumor microenvironment remodeling. In coronary artery disease (CAD), macrophages accumulate lipids to form foam cells, secrete pro-inflammatory cytokines, and contribute to plaque instability and necrotic core expansion. In malignancy, tumor-associated macrophages (TAMs) support angiogenesis, immune suppression, extracellular matrix remodeling, and metastatic dissemination (106–110). Despite differences in tissue context, macrophages in both settings rely on overlapping metabolic programs, including enhanced glycolysis, lipid uptake, mitochondrial remodeling, reactive oxygen species (ROS) production, and inflammasome activation (111–114). The immunometabolic phenotype of macrophages is influenced by genetic and environmental factors. Clonal hematopoiesis provides one prominent example. Fidler et al. demonstrated that JAK2V617F-mutant macrophages exhibit increased glycolytic activity, leading to DNA replication stress and activation of the AIM2 inflammasome (64). This metabolic rewiring accelerates vascular inflammation and plaque progression, providing a direct link between hematopoietic mutations, immune-cell metabolism, and CAD pathology. Similarly, Yalcinkaya et al. showed that TET2-mutant macrophages under hypercholesterolemic conditions activate the NLRP3 inflammasome through BRCC3-mediated deubiquitylation (65). This study highlights the interplay between CHIP genotype, metabolic stress, and inflammasome-driven vascular inflammation.

These findings illustrate that metabolic reprogramming of macrophages is a shared mechanism in CAD and cancer. In tumors, macrophages exposed to hypoxic, nutrient-depleted, or lactate-rich microenvironments undergo similar glycolytic shifts and mitochondrial remodeling. These adaptations enable TAMs to survive under metabolic stress, produce immunosuppressive cytokines, and support tumor growth. Therefore, macrophage metabolic plasticity provides a molecular link between cardiovascular inflammation and cancer-promoting immune microenvironments.

### 5.2 T-cell metabolism and immune tolerance failure

While myeloid cells provide the initial inflammatory drive, adaptive immune cells, particularly T cells, are critical mediators of both atherosclerosis and tumor immunity. In CAD, T cells infiltrate atherosclerotic plaques where they can undergo clonal expansion in response to local antigenic stimuli (115–120). Single-cell RNA sequencing and T-cell receptor (TCR) profiling studies indicate that breakdown of T-cell tolerance checkpoints occurs within plaques, leading to the expansion of autoreactive or pro-inflammatory T-cell clones that further exacerbate vascular inflammation.

Chowdhury et al. demonstrated that human coronary plaque T cells are clonally expanded and can cross-react with viral antigens as well as self-antigens, providing direct evidence for antigen-driven T-cell responses in atherosclerosis (121). Wang et al. further revealed, using single-cell RNA and TCR profiling, that loss of T-cell tolerance checkpoints within plaques contributes to chronic inflammatory signaling (122). This phenomenon closely mirrors T-cell dysfunction in cancer, where clonal expansion, immune checkpoint regulation, and exhaustion dictate both tumor progression and therapeutic response. In addition, the plaque immune microenvironment is shaped by metabolic stressors such as hypoxia, lipid accumulation, and oxidative stress. These conditions can alter T-cell metabolism, promoting a shift toward glycolysis, fatty acid oxidation, or mitochondrial dysfunction, which in turn impacts effector function and immune tolerance. Durán et al. provided a detailed map of immune checkpoint expression in human atherosclerotic plaques and demonstrated that cardiometabolic factors modulate T-cell checkpoint signaling (123). This work underscores the role of immune-metabolic crosstalk in bridging cardiovascular inflammation and cancer-relevant immune dysregulation.

### 5.3 Immune costimulatory pathways shared by cancer and CAD

Beyond intrinsic metabolic shifts, immune costimulatory and checkpoint pathways orchestrate the activation and function of both innate and adaptive immune cells in CAD and malignancy. The CD40L–CD40 axis is particularly relevant because it participates simultaneously in antigen presentation, T-cell activation, macrophage polarization, endothelial inflammation, platelet activation, and plaque progression. Dysregulation of this axis can exacerbate vascular inflammation while also influencing tumor immunity and the balance between antitumor responses and immune suppression. Lacy et al. demonstrated that CD40L–CD40 signaling has cell-type-specific and divergent roles in atherosclerotic vascular disease (124). For instance, activation in macrophages promotes pro-inflammatory cytokine production and foam-cell formation, whereas endothelial cell CD40 signaling regulates adhesion molecule expression and leukocyte recruitment. This cellular heterogeneity may explain why global targeting of CD40L–CD40 has complex outcomes and highlights the need for precision strategies that account for cell-specific effects in both CAD and cancer.

**Figure 5:**
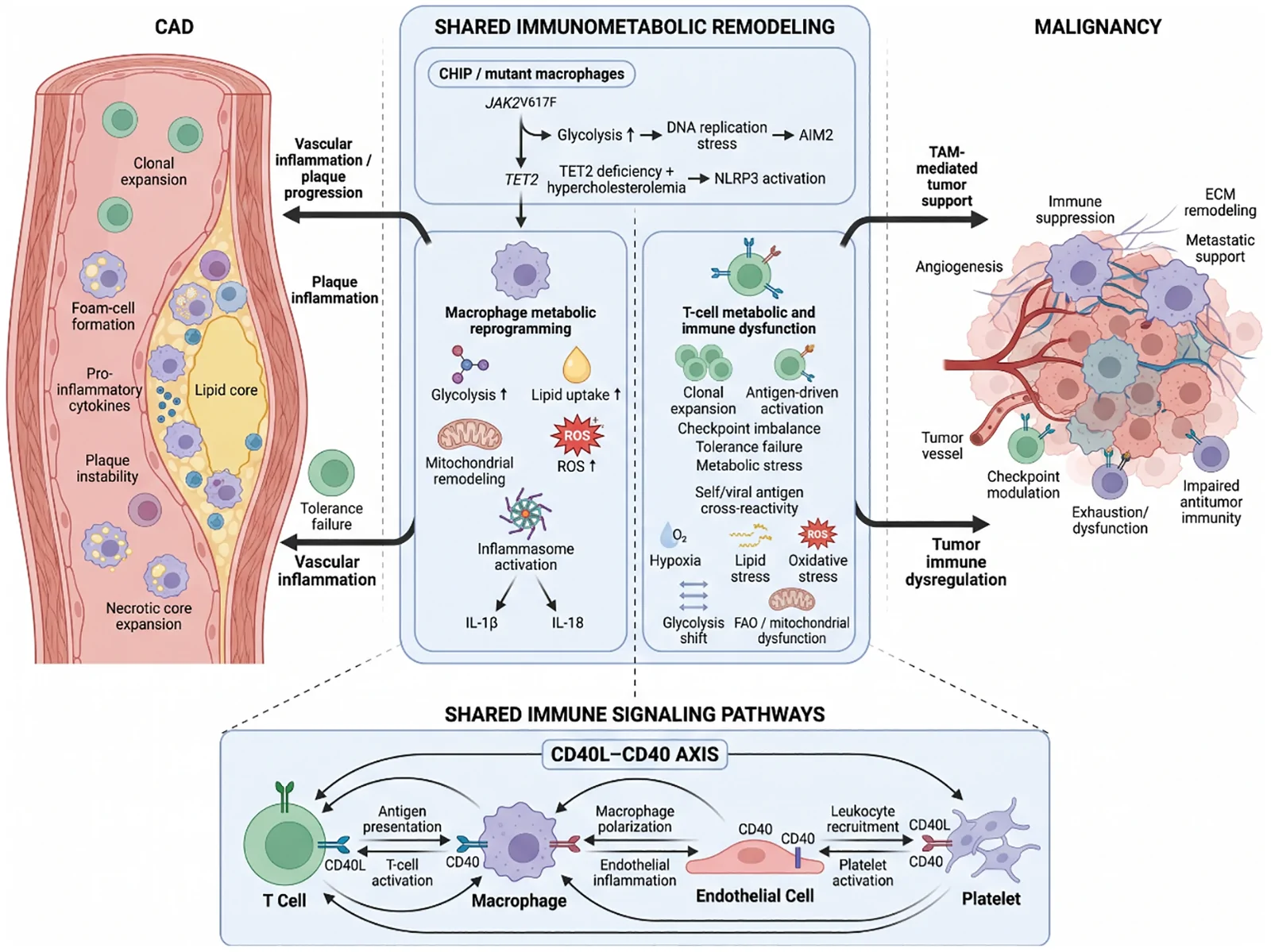
Immunometabolic remodeling links CAD and malignancy through shared macrophage and T-cell programs. In atherosclerotic plaques, metabolically reprogrammed macrophages promote lipid uptake, foam-cell formation, inflammasome activation, and plaque instability. In tumors, TAMs support angiogenesis, immune suppression, and metastasis. T-cell clonal expansion, tolerance failure, checkpoint imbalance, and CD40L–CD40 signaling further amplify vascular inflammation and tumor immune dysregulation.

Taken together, these studies indicate that immunometabolic remodeling is a shared pathogenic mechanism linking CAD and malignancy. Metabolically reprogrammed macrophages, antigen-driven T cells, and costimulatory pathways such as CD40L–CD40 collectively create an environment in which chronic inflammation, immune dysregulation, and vascular injury converge with tumor-promoting immune dysfunction. By integrating myeloid and lymphoid metabolic adaptations with costimulatory signaling, this framework explains how immune metabolism serves as a bridge between cardiovascular and oncologic pathophysiology. **Figure 5** illustrates how macrophage and T-cell metabolic reprogramming creates a shared immune mechanism connecting atherosclerotic plaque progression with tumor microenvironment remodeling. It highlights CHIP-related inflammasome activation, T-cell tolerance failure, and CD40L–CD40 signaling as key immunometabolic bridges between CAD and malignancy.

## 6 Thrombosis, platelet activation, endothelial dysfunction, and NETs

### 6.1 Cancer-associated thrombosis as a shared vascular mechanism

Cancer-associated thrombosis represents a major clinical and mechanistic intersection between malignancy and cardiovascular disease (125–127). Traditionally regarded as a secondary complication of cancer, thrombosis in the oncologic context is increasingly recognized as an active contributor to tumor progression and metastasis. The mechanisms underlying cancer-associated thrombosis share remarkable similarities with the pathways driving coronary artery disease (CAD). Both conditions involve platelet activation, endothelial injury, tissue factor signaling, neutrophil activation, and coagulation cascade engagement. However, thrombotic phenotypes in CAD and malignancy should not be treated as biologically identical. In CAD, arterial thrombosis usually develops on a background of atherosclerotic plaque inflammation, plaque rupture or erosion, platelet activation, and coronary flow limitation. In cancer, thrombotic manifestations more often include venous thromboembolism, tumor-associated hypercoagulability, microvascular thrombosis, and metastatic tumor-cell arrest, although arterial events may also occur in selected patients. Therefore, platelet activation, coagulation, endothelial activation, and NET formation should be interpreted as partially overlapping but phenotype-dependent mechanisms rather than as uniform drivers of all thrombotic events.

Experimental evidence highlights the therapeutic and mechanistic relevance of these pathways. Palacios-Acedo et al. demonstrated that P2RY12 inhibition not only reduces cancer-associated thrombosis but also limits pancreatic tumor growth, indicating that platelet activation directly contributes to tumor progression (128). Similarly, Sano et al. showed that blockade of VCAM-1 attenuates pancreatic tumor progression and diminishes cancer-associated thrombosis or thromboembolism, highlighting endothelial adhesion molecules as central mediators linking tumor biology and vascular pathology (129). Feinauer et al. provided further mechanistic evidence, showing that localized activation of coagulation within the microvasculature facilitates tumor cell arrest and metastatic seeding in the brain, thereby demonstrating the direct role of thromboinflammation in promoting metastasis (130). These studies collectively illustrate that thrombosis in cancer is not merely a passive epiphenomenon but an active biological interface that mirrors processes observed in CAD. Both diseases exploit coagulation and platelet-mediated pathways to promote tissue injury, inflammatory amplification, and microvascular obstruction.

### 6.2 Platelet–tumor cell crosstalk

Platelets act as critical mediators of this thromboinflammatory interface. By interacting with tumor cells through surface adhesion molecules, soluble mediators, and coagulation factors, platelets can shield malignant cells from immune surveillance, facilitate adhesion to endothelial surfaces, and promote metastatic dissemination. This crosstalk creates a dual role for platelets in both CAD and malignancy: as contributors to vascular occlusion and as facilitators of tumor progression. Palacios-Acedo et al. highlighted the translational relevance of this interaction by demonstrating that pharmacological inhibition of P2RY12 reduces both platelet-mediated thrombosis and tumor growth in pancreatic cancer models (128). Furthermore, in a comparative analysis, Palacios-Acedo et al. showed that the platelet–tumor cell interaction contributes to cancer type-specific differences in thrombosis risk, suggesting that certain malignancies exploit platelet-mediated pathways more effectively than others (131). These findings underscore the importance of considering platelet biology in both cardiovascular and oncologic contexts, particularly when designing antiplatelet therapies for patients with CAD who are also at risk for malignancy.

### 6.3 Endothelial activation and adhesion molecules

Endothelial dysfunction serves as a central node connecting CAD and cancer-associated thrombosis. Activated endothelium expresses adhesion molecules such as VCAM-1, ICAM-1, and E-selectin, which facilitate leukocyte recruitment, platelet adhesion, and tumor–endothelium interactions. Endothelial activation amplifies inflammatory signaling and promotes local thrombus formation, creating a permissive microenvironment for both plaque destabilization in CAD and metastatic seeding in cancer. Sano et al. demonstrated that VCAM-1 blockade reduces pancreatic tumor progression and diminishes thrombosis, illustrating the dual relevance of endothelial adhesion molecules in both vascular and oncologic pathophysiology (129). Endothelial activation, therefore, serves as a critical mechanistic bridge that links systemic inflammation, thrombosis, and tumor progression, while simultaneously mediating cardiovascular risk.

### 6.4 NETs as inflammatory scaffolds for thrombosis

Neutrophil extracellular traps (NETs) provide a structural and functional scaffold for thrombus formation by promoting platelet adhesion, coagulation activation, and fibrin deposition. NETs are not only central to the propagation of cancer-associated thrombosis but also contribute to tumor progression and the establishment of metastatic niches. By capturing circulating tumor cells and localizing thromboinflammatory signaling, NETs integrate immune, vascular, and hemostatic pathways in a manner that mirrors mechanisms observed in CAD. Li et al. demonstrated that NET formation in gastric cancer patients contributes to cancer-associated thrombosis, highlighting the pathological crosstalk between neutrophils, platelets, and tumor cells (132). Feinauer et al. further showed that local coagulation facilitates tumor cell arrest in brain vasculature, reinforcing the concept that thromboinflammation serves as a mechanistic interface linking malignancy and cardiovascular events (130). Collectively, these studies indicate that NETs, by coordinating immune and coagulation pathways, represent a shared molecular mechanism underlying both CAD and cancer-associated thrombotic risk.

**Figure 6:**
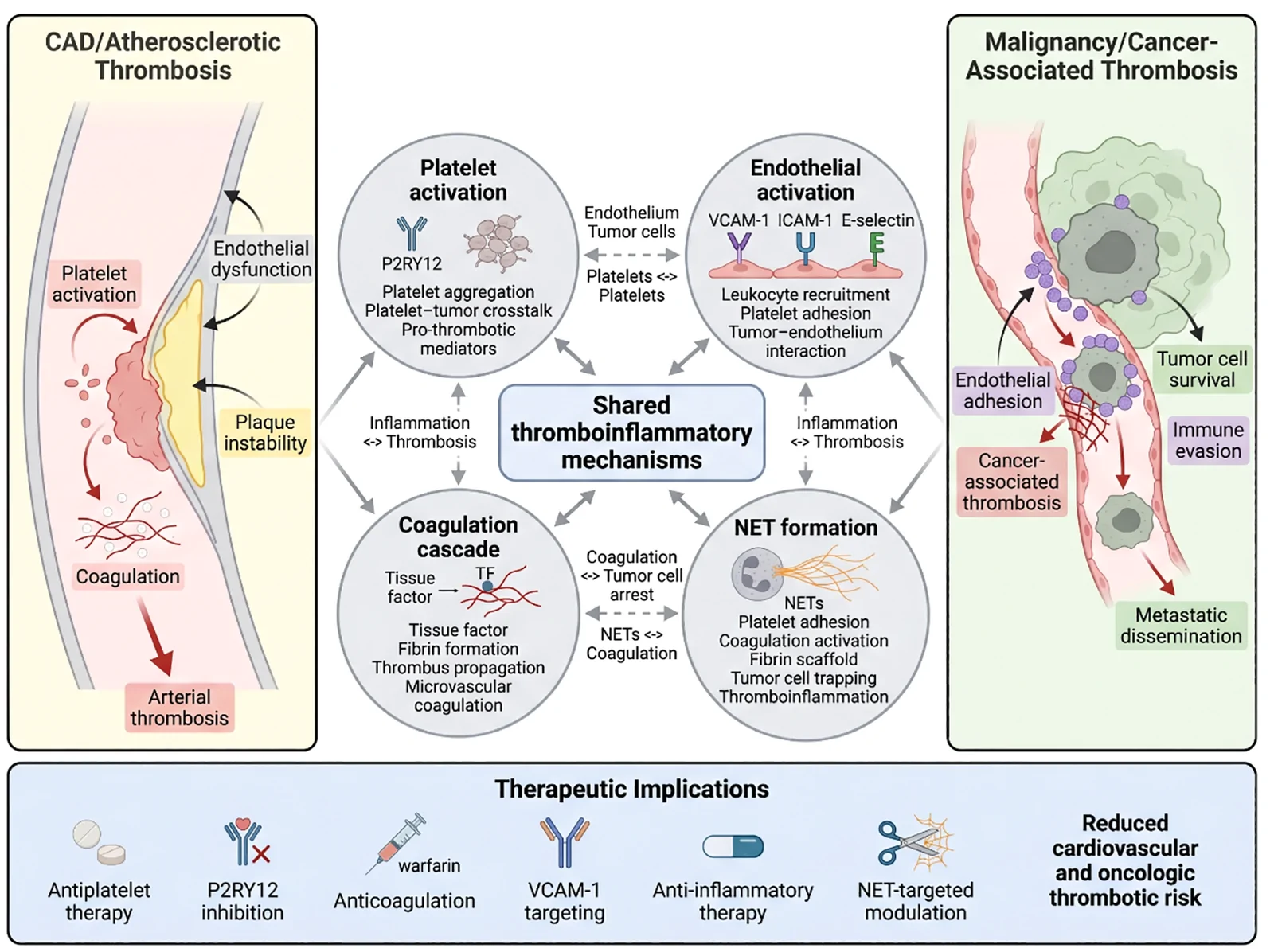
Thromboinflammatory mechanisms linking CAD and malignancy. Platelet activation, endothelial dysfunction, coagulation cascade activation, and NET formation jointly promote plaque instability, arterial thrombosis, cancer-associated thrombosis, tumor cell survival, endothelial adhesion, and metastatic dissemination. Platelet–tumor cell crosstalk, VCAM-1-mediated endothelial activation, and NET-dependent fibrin scaffolds represent shared vascular mechanisms and potential therapeutic targets for antiplatelet, anticoagulant, endothelial-directed, and anti-inflammatory interventions.

Taken together, platelet activation, coagulation, endothelial dysfunction, and NET formation provide partially overlapping thromboinflammatory links between CAD and malignancy. However, these mechanisms operate differently across thrombotic phenotypes. In CAD, they most prominently contribute to plaque inflammation, plaque rupture or erosion, arterial thrombosis, and acute coronary events. In malignancy, they more often participate in venous thromboembolism, tumor-associated hypercoagulability, microvascular thrombosis, immune evasion, and metastatic tumor-cell arrest. Therefore, antiplatelet, anticoagulant, endothelial-directed, or NET-targeted strategies should be considered according to the dominant thrombotic phenotype rather than generalized across all patients with CAD–malignancy comorbidity(**Table 3**). **Figure 6** illustrates how thrombosis-related pathways form a shared vascular interface between CAD and malignancy. It highlights platelets, activated endothelium, coagulation, and NETs as interconnected mechanisms driving both cardiovascular events and cancer progression.

**Table 3:** Thrombotic phenotypes and dominant thromboinflammatory mechanisms in CAD–malignancy comorbidity.

Mechanistic axis	Major role in CAD	Major role in malignancy	Dominant thrombotic phenotype	Key implication
Platelet-driven mechanisms	Platelet adhesion, activation, and aggregation contribute to arterial thrombosis after plaque rupture or erosion and drive acute coronary syndromes.	Platelets shield circulating tumor cells, promote tumor–endothelium adhesion, support immune evasion, and facilitate metastatic dissemination.	CAD: arterial thrombosis and acute coronary events. Cancer: tumor-cell dissemination and selected arterial or venous thrombotic events.	Antiplatelet strategies may have dual relevance, but benefit depends on coronary risk, tumor type, platelet count, and bleeding risk.
Coagulation-driven mechanisms	Coagulation cascade activation contributes to fibrin-rich thrombus formation after plaque disruption and may worsen coronary occlusion.	Tissue factor expression, thrombin generation, and systemic hypercoagulability drive cancer-associated venous thromboembolism and microvascular thrombosis.	CAD: plaque-associated coronary thrombosis. Cancer: venous thromboembolism, microvascular thrombosis, and metastatic vascular niche formation.	Anticoagulant approaches are more directly relevant to cancer-associated VTE than to all CAD-related thrombotic events.
Endothelial-driven mechanisms	Endothelial dysfunction promotes leukocyte recruitment, platelet adhesion, vasomotor dysfunction, and plaque progression or destabilization.	Activated tumor-associated endothelium supports tumor-cell adhesion, vascular permeability, angiogenesis, thrombosis, and metastatic seeding.	CAD: endothelial inflammation, plaque progression, and plaque destabilization. Cancer: tumor–endothelium interaction and microvascular arrest.	Endothelial biomarkers and vascular imaging may help identify patients with active vascular inflammation.
NET-driven mechanisms	NETs amplify thromboinflammation by promoting platelet adhesion, coagulation activation, fibrin deposition, and plaque instability.	NETs support cancer-associated thrombosis, capture circulating tumor cells, and contribute to metastatic niche formation.	CAD: plaque inflammation and coronary thrombus amplification. Cancer: VTE, microvascular thrombosis, and metastatic tumor-cell trapping.	NET-rich phenotypes may represent a distinct thromboinflammatory endotype rather than a universal feature of all thrombotic events.

## 7 Adaptive immunity, immune checkpoints, and atherosclerotic plaque biology

### 7.1 Coronary plaques as immune-active niches

Atherosclerotic plaques are increasingly recognized as immune-active tissue niches rather than passive lipid deposits. In addition to lipid accumulation and vascular smooth muscle cell remodeling, plaques contain diverse immune and stromal components, including macrophages, dendritic cells, T cells, B cells, plasma cells, endothelial cells, fibroblast-like cells, and extracellular matrix-rich regions. These cellular populations interact dynamically to shape plaque progression, inflammatory amplification, and plaque instability. This concept is highly relevant to CAD–malignancy comorbidity because tumors are also organized immune ecosystems in which immune-cell localization, antigen recognition, checkpoint signaling, and stromal organization determine disease behavior (133–135).

Adaptive immunity plays a particularly important role in the chronicity and specificity of atherosclerotic inflammation. Unlike acute innate immune activation, T-cell responses can persist, expand clonally, and acquire memory-like features. The presence of clonally expanded T cells within coronary plaques suggests that plaque inflammation may be driven, at least in part, by antigen-specific immune responses rather than nonspecific inflammation alone. This creates an important conceptual parallel between atherosclerotic plaques and tumors, both of which can contain clonally expanded lymphocytes shaped by local antigenic stimulation.

Chowdhury et al. provided strong evidence for this concept by demonstrating that human coronary plaque T cells are clonal and can cross-react with viral and self-antigens (121). This finding suggests that coronary plaques may harbor antigen-experienced T-cell populations capable of recognizing both exogenous and endogenous antigens. Such cross-reactivity could contribute to persistent vascular inflammation, especially in patients with chronic infections, immune aging, or autoimmune-like immune activation. In the context of CAD–malignancy comorbidity, this observation is important because cancer also depends heavily on antigen-specific T-cell recognition, clonal expansion, and immune escape. Wang et al. further expanded this framework by combining single-cell RNA analysis with T-cell antigen receptor profiling (122). Their study indicated that T-cell tolerance checkpoints are disrupted in atherosclerosis, allowing pathogenic or autoreactive T-cell responses to persist within vascular lesions. This breakdown of tolerance may help explain why atherosclerosis behaves as a chronic inflammatory disease even in the absence of active infection. It also suggests that plaque immunity may be sensitive to systemic immune perturbations, including cancer-associated inflammation and cancer immunotherapy.

Together, these studies support the view that coronary plaques are immunologically structured lesions characterized by antigen presentation, T-cell clonal expansion, and defective immune tolerance. This perspective provides a mechanistic bridge to cancer immunology, where similar principles govern tumor immune surveillance, immune escape, and response to therapy. Therefore, adaptive immune regulation within plaques should be considered an important component of CAD–malignancy comorbidity.

### 7.2 Immune checkpoint regulation in atherosclerosis

Immune checkpoints are best known for their roles in cancer, where they regulate T-cell activation, exhaustion, tolerance, and antitumor immunity (136–138). However, immune checkpoint pathways are not restricted to tumors. They also operate in chronically inflamed tissues, including atherosclerotic plaques, where they help restrain excessive immune activation and maintain local immune homeostasis. In this context, immune checkpoints may function as protective brakes that limit tissue damage within the vascular wall. The relevance of immune checkpoints to CAD–malignancy comorbidity has become increasingly important with the widespread use of immune checkpoint inhibitors (ICIs) in oncology. ICIs can restore antitumor T-cell activity by blocking inhibitory pathways such as PD-1, PD-L1, and CTLA-4. However, the same therapeutic principle that enhances antitumor immunity may also disturb immune balance in cardiovascular tissues. If atherosclerotic plaques rely on checkpoint pathways to suppress pathogenic T-cell activation, systemic checkpoint blockade may increase plaque inflammation, promote vascular immune activation, or contribute to cardiovascular toxicity in susceptible patients.

Durán et al. provided important evidence by defining the immune checkpoint landscape of human atherosclerosis (123). Their study showed that human atherosclerotic plaques contain a complex network of immune checkpoint molecules, and that this landscape is influenced by cardiometabolic factors. This finding has two major implications. First, atherosclerotic plaques possess endogenous checkpoint-regulated immune circuits, indicating that vascular inflammation is actively restrained by immune regulatory pathways. Second, metabolic conditions such as obesity, diabetes, and dyslipidemia may modulate checkpoint expression within plaques, potentially altering the vulnerability of patients to immune-mediated cardiovascular injury. The findings of Wang et al. further support this concept by showing that T-cell tolerance checkpoints are disrupted in atherosclerosis (122). This suggests that plaque inflammation may arise not only from increased antigenic stimulation but also from insufficient immune restraint. In cancer, loss or therapeutic blockade of immune checkpoints can reinvigorate T-cell responses against tumor cells. In atherosclerosis, however, excessive T-cell activation may aggravate vascular inflammation and plaque instability. Therefore, the biological consequences of checkpoint modulation depend strongly on tissue context.

This checkpoint-centered view has major implications for cardio-oncology. Cancer patients with pre-existing CAD, high plaque burden, diabetes, dyslipidemia, smoking history, or clonal hematopoiesis may already have inflamed vascular lesions. In these individuals, ICI therapy could theoretically remove critical inhibitory signals from plaque-infiltrating T cells, thereby exacerbating vascular inflammation. Although the precise contribution of checkpoint blockade to CAD progression requires further investigation, the presence of a defined checkpoint landscape in human plaques provides a plausible mechanistic basis for ICI-associated cardiovascular complications. Thus, immune checkpoint regulation represents a direct mechanistic intersection between tumor immunotherapy and atherosclerotic disease. Understanding how checkpoint pathways function in vascular tissues may help identify patients at increased cardiovascular risk during immunotherapy and guide the development of monitoring strategies that extend beyond myocarditis alone to include plaque inflammation and CAD progression.

### 7.3 CD40L–CD40 as a shared immune activation pathway

In addition to inhibitory immune checkpoints, costimulatory pathways are central regulators of immune activation in both cancer and CAD. Among these, the CD40L–CD40 axis is particularly relevant because it connects adaptive immunity, innate immunity, endothelial activation, platelet activation, and vascular inflammation. CD40 is expressed on antigen-presenting cells, endothelial cells, smooth muscle cells, and other stromal or immune populations, while CD40L is expressed on activated T cells and platelets (139–142). Through these interactions, CD40L–CD40 signaling coordinates immune-cell activation and vascular inflammatory responses.

In cancer, CD40 signaling can influence antigen presentation, dendritic cell activation, T-cell priming, macrophage polarization, and antitumor immunity. Depending on context, CD40 activation may enhance immune-mediated tumor control or contribute to inflammation-associated tumor progression. In CAD, CD40L–CD40 signaling promotes endothelial activation, adhesion molecule expression, leukocyte recruitment, macrophage cytokine production, matrix remodeling, and plaque progression. Therefore, this axis is a shared immunovascular pathway that links tumor immunity with atherosclerotic inflammation. Lacy et al. showed that CD40L–CD40 signaling has cell-specific and divergent roles in atherosclerotic vascular disease (124). This finding is important because it indicates that the CD40L–CD40 axis cannot be understood as a uniformly harmful or uniformly protective pathway. Its biological effects depend on the cell type, disease stage, and local inflammatory context. For example, CD40 signaling in endothelial cells may promote leukocyte adhesion and vascular inflammation, while CD40 signaling in antigen-presenting cells may shape T-cell responses. Platelet-derived CD40L may further contribute to thromboinflammation, linking adaptive immune activation with coagulation and thrombosis.

This cell-specific complexity has important translational implications. Global inhibition of CD40L–CD40 signaling may reduce vascular inflammation but could also impair immune defense or antitumor immunity. Conversely, selective modulation of specific CD40-expressing cell populations may provide a more precise strategy. In CAD–malignancy comorbidity, this is especially relevant because patients may require preservation of antitumor immune responses while limiting vascular inflammation and thrombotic risk. The CD40L–CD40 pathway also provides a conceptual bridge between Sections 6 and 7 of this review. Platelets, endothelial cells, macrophages, and T cells all participate in CD40L–CD40 signaling. Thus, this axis links platelet activation and thrombosis with adaptive immune activation and plaque inflammation. It helps explain how immune costimulation, endothelial dysfunction, and thromboinflammation can converge in patients with cancer and CAD.

**Figure 7:**
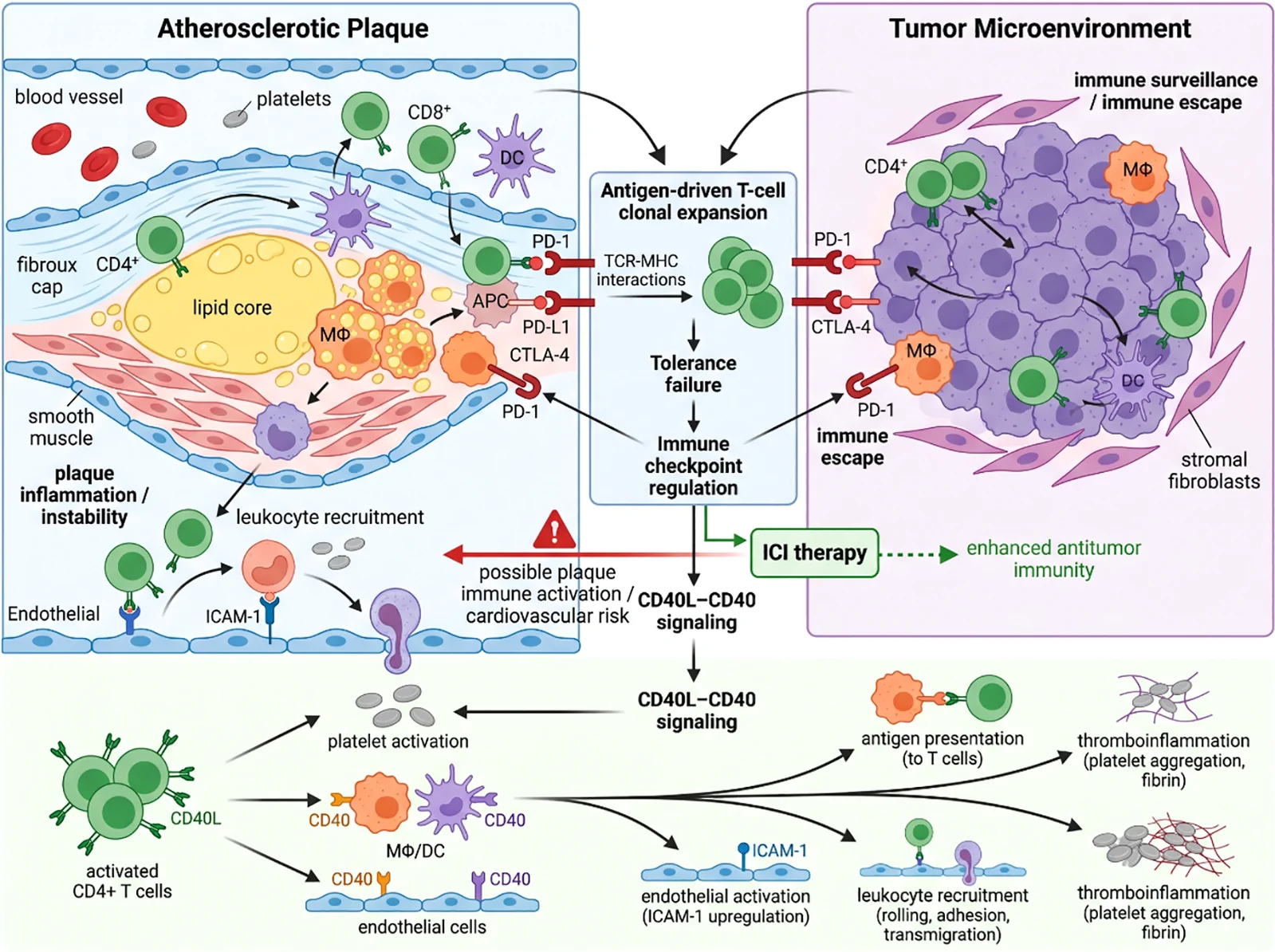
Adaptive immune mechanisms linking atherosclerotic plaques and malignancy. Coronary plaques and tumors both contain antigen-experienced immune cells shaped by T-cell clonal expansion, tolerance failure, immune checkpoint regulation, and CD40L–CD40 signaling. Checkpoint pathways may restrain plaque inflammation while supporting tumor immune escape, whereas immune checkpoint blockade can enhance antitumor immunity but may also promote vascular immune activation and cardiovascular risk.

In summary, adaptive immunity is a central component of atherosclerotic plaque biology and a major mechanistic bridge to cancer immunology. Coronary plaques contain clonally expanded and antigen-experienced T cells, display evidence of tolerance checkpoint disruption, and express immune checkpoint and costimulatory pathways that regulate vascular inflammation. These features make plaques immunologically dynamic lesions that may be affected by systemic inflammation, malignancy, and cancer immunotherapy. Understanding these adaptive immune circuits is essential for developing cardio-oncology strategies that preserve antitumor immunity while preventing immune-mediated vascular injury. The major molecular mechanisms linking CAD and malignancy converge on inflammation, clonal hematopoiesis, inflammasome activation, immune-cell metabolic remodeling, thromboinflammation, and immune checkpoint regulation. These shared pathways and their representative supporting studies are summarized in **Table 4**. **Figure 7** illustrates how atherosclerotic plaques and tumors share adaptive immune circuits involving T-cell clonality, checkpoint regulation, and CD40L–CD40 signaling. It highlights the potential cardio-oncology consequence that immune checkpoint blockade may disturb plaque immune homeostasis while restoring antitumor immunity.

**Table 4:** Mechanistic pathways and molecular mediators of CAD–malignancy comorbidity.

Pathway/process	Key mediators	Evidence/mechanism	Relevant studies (PMID)
Chronic inflammation	IL-1β, IL-6, TNF-α, CRP	Promotes endothelial activation, plaque progression, tumor initiation, angiogenesis, immune suppression	Sun M (38267838), Zhao YW (36339468)
Clonal hematopoiesis	TET2, DNMT3A, JAK2, ASXL1	CHIP-mutant myeloid cells drive vascular inflammation and predispose to malignancy	Díez-Díez M (39215150), Fidler TP (33731931), Yalcinkaya M (37781816)
Inflammasome activation	AIM2, NLRP3	Drives macrophage inflammatory responses, endothelial injury, and atherosclerosis	Fidler TP (33731931), Yalcinkaya M (37781816)
Immunometabolism	Glycolysis, lipid uptake, mitochondrial dysfunction	Metabolically reprogrammed macrophages and T cells amplify vascular inflammation and tumor-supportive immunity	Chowdhury RR (35430876), Wang Z (37621765)
Thrombosis/NETs	Platelets, VCAM-1, tissue factor, NETs	Tumor-induced thrombosis, vascular occlusion, metastatic niche formation, plaque instability	Palacios-Acedo AL (34589424, 35159000), Li JC (36051331), Feinauer MJ (33270819)
Immune checkpoints	PD-1, PD-L1, CTLA-4, CD40L–CD40	Regulate T-cell activation in plaques and tumors; ICI therapy may perturb vascular immune homeostasis	Durán JGB (39613875), Lacy M (34145241)

## 8 Cardio-oncology translation: cancer therapy, CAD progression, and cardiovascular immune toxicity

### 8.1 Immune checkpoint inhibitors: myocarditis, accelerated atherosclerosis, and coronary events check

Importantly, ICI-associated cardiovascular toxicity should not be considered a single mechanistic entity. ICI-associated myocarditis, accelerated atherosclerosis, and acute coronary events may overlap clinically but differ biologically (143, 144). ICI-associated myocarditis primarily reflects immune-mediated myocardial injury, in which checkpoint blockade disrupts peripheral tolerance and allows autoreactive T cells to recognize cardiac antigens, resulting in myocardial inflammation, troponin elevation, arrhythmias, heart failure, or fulminant myocarditis. Zhu et al. identified pathogenic immune-cell subsets that drive checkpoint inhibitor-induced myocarditis, including pro-inflammatory T-cell populations capable of tissue infiltration and cytokine production (145). Complementing this, Axelrod et al. demonstrated that α-myosin-specific CD8^+^ T cells are sufficient to mediate ICI-associated myocarditis in preclinical models (146). By contrast, ICI-associated accelerated atherosclerosis is more closely related to vascular immune activation, plaque inflammation, macrophage and T-cell activation within atherosclerotic lesions, and loss of checkpoint-mediated immune restraint in the arterial wall. Patients with pre-existing CAD or subclinical atherosclerosis may therefore experience exacerbated plaque inflammation or accelerated vascular injury when inhibitory checkpoints are blocked. Durán et al. mapped the immune checkpoint landscape in human atherosclerotic plaques and showed that checkpoint molecules are highly expressed in plaque-infiltrating immune cells and modulated by cardiometabolic factors (123). Acute coronary events, including myocardial infarction or unstable angina, may represent downstream clinical outcomes of plaque inflammation, plaque rupture or erosion, coronary thrombosis, endothelial dysfunction, or supply–demand mismatch. Therefore, myocarditis should be discussed as immune-mediated myocardial injury, whereas ICI-associated atherosclerotic events should be interpreted within the framework of plaque immune dysregulation and accelerated atherosclerosis.

### 8.2 Autoantigen-specific T-cell responses and cardiovascular injury

One key mechanism by which ICIs can cause cardiovascular injury is the unmasking of autoreactive T-cell responses against cardiovascular antigens. This phenomenon has been most extensively studied in ICI-associated myocarditis but has implications for plaque inflammation and atherosclerotic disease. In the myocarditis model, α-myosin-specific T cells clonally expand and infiltrate cardiac tissue, mediating cytotoxic damage and inflammatory cytokine release (146). Zhu et al. further identified specific immune-cell subsets within the myocardium that contribute to tissue inflammation during ICI therapy (145). These observations indicate that checkpoint blockade can disrupt previously maintained tolerance to self-antigens, enabling pathogenic adaptive immune responses. Although direct studies in coronary plaques are limited, similar mechanisms may operate in the context of atherosclerotic lesions, where autoreactive T cells recognize plaque antigens and contribute to inflammation and instability.

### 8.3 Cardiometabolic and immune checkpoint interactions in plaques

Atherosclerotic plaques themselves are not immunologically inert. They express immune checkpoint molecules that regulate T-cell and myeloid cell activation, contributing to local immune homeostasis. Durán et al. demonstrated that these checkpoints are dynamically influenced by cardiometabolic factors such as diabetes, obesity, dyslipidemia, and smoking (123). In patients with established CAD, pre-existing plaque immune checkpoints may provide a protective brake on pathogenic T-cell activation. Checkpoint blockade in this context could remove these inhibitory signals, potentially exacerbating vascular inflammation and plaque destabilization. Wang et al. further showed that tolerance checkpoints are disrupted in atherosclerosis, supporting the notion that cardiovascular immune regulation is sensitive to systemic immune perturbations (122). Taken together, these studies suggest that ICIs, while promoting antitumor immunity, may also destabilize vascular immune homeostasis, highlighting the mechanistic intersection between cancer therapy and CAD progression.

### 8.4 Toward precision cardio-oncology

The complex interplay between systemic inflammation, CHIP, thrombosis, immune checkpoints, and cardiovascular risk underscores the need for a precision cardio-oncology approach. Future management strategies should integrate multiple layers of patient-specific information, including inflammatory biomarkers, CHIP mutation status, thrombotic risk, coronary plaque burden, cancer type, cancer therapy exposure, and immune checkpoint landscape. Zuriaga et al. demonstrated that CHIP-guided anti-inflammatory therapy, such as colchicine in TET2-mutant clonal hematopoiesis, can mitigate vascular risk (71). Palacios-Acedo et al. showed that P2RY12 inhibition reduces both cancer-associated thrombosis and tumor progression, highlighting the dual benefit of antiplatelet therapy in cardio-oncology (128). Sano et al. further demonstrated that VCAM-1 blockade can reduce tumor growth and associated thrombosis, emphasizing endothelial-mediated contributions to CAD–malignancy comorbidity (129). Durán et al. provide a blueprint for mapping immune checkpoint expression within plaques to assess ICI-related risk (123).

In practical terms, a precision cardio-oncology strategy could involve comprehensive patient profiling and individualized intervention. Systemic inflammatory burden may be assessed through biomarkers such as CRP, IL-6, IL-1β, and the neutrophil-to-lymphocyte ratio, while genotyping for clonal hematopoiesis of indeterminate potential (CHIP) can identify hematopoietic clones that increase susceptibility to both cardiovascular inflammation and malignancy. Risk of thrombosis and platelet activation can be evaluated through coagulation parameters and platelet function testing, and integration of coronary imaging with plaque characterization and immune mapping—including assessment of immune checkpoint expression—can identify patients at higher risk of therapy-induced vascular injury. Together, these insights can guide tailored pharmacologic interventions, including targeted anti-inflammatory, antiplatelet, or endothelial-modulatory therapies, designed to protect cardiovascular health without compromising antitumor efficacy.

By combining these layers of patient-specific information, clinicians may better anticipate and mitigate cardiovascular complications in patients undergoing cancer therapy, while preserving antitumor responses and improving overall outcomes. This integrated approach embodies the emerging field of precision cardio-oncology, which seeks to harmonize cardiovascular and oncologic care in patients at the intersection of these high-risk diseases.

## 9 Therapeutic implications

### 9.1 Anti-inflammatory strategies

Because chronic inflammation is a central mechanism linking coronary artery disease (CAD) and malignancy, anti-inflammatory therapy represents an important translational opportunity in CAD–malignancy comorbidity. This strategy may be particularly relevant for patients with persistent systemic inflammation, elevated inflammatory biomarkers, clonal hematopoiesis of indeterminate potential (CHIP), recurrent cardiovascular events despite lipid lowering, or cancer-associated inflammatory states. Rather than applying anti-inflammatory treatment indiscriminately, future approaches should aim to identify molecularly defined inflammatory endotypes that are most likely to benefit.

CHIP-associated inflammation provides one of the clearest examples of mechanism-guided anti-inflammatory intervention. Zuriaga et al. demonstrated that colchicine prevents accelerated atherosclerosis in TET2-mutant clonal hematopoiesis (71). This finding suggests that the cardiovascular consequences of mutant hematopoietic clones may be therapeutically modifiable. In patients with cancer or cancer survivors, where CHIP prevalence increases with age and prior therapy exposure, CHIP-guided anti-inflammatory prevention may become an important component of cardio-oncology risk management. Inflammasome inhibition represents another promising direction. Fidler et al. showed that JAK2V617F clonal hematopoiesis accelerates atherosclerosis through macrophage glycolysis, DNA replication stress, and AIM2 inflammasome activation (64). This identifies AIM2 as a potential mechanistic target in selected CHIP-associated vascular inflammation. Similarly, Yalcinkaya et al. demonstrated that TET2 clonal hematopoiesis promotes atherosclerosis through JNK1–BRCC3-mediated NLRP3 deubiquitylation and inflammasome activation, highlighting NLRP3 regulation as another actionable inflammatory node (65).

Clinically, anti-inflammatory therapy in CAD–malignancy comorbidity must be carefully balanced. Although chronic inflammation can promote atherosclerosis, tumor progression, and thromboinflammation, broad inflammatory suppression should not be assumed to be uniformly beneficial in patients with cancer. Inflammation also supports antigen presentation, dendritic-cell activation, T-cell priming, and antitumor immune surveillance; therefore, indiscriminate inhibition of inflammatory pathways may impair host defense or weaken responses to immunotherapy in selected tumor contexts (147–149). A more appropriate strategy is to define inflammatory endotypes that may benefit from targeted intervention. For example, patients with residual systemic inflammation and elevated hsCRP or IL-6 after optimal lipid-lowering therapy may represent candidates for IL-1β/IL-6-axis modulation; patients with TET2-mutant CHIP, myeloid inflammatory remodeling, or IL-1β/NLRP3-related signatures may be more suitable for colchicine- or inflammasome-directed approaches; and patients with NET-rich thromboinflammatory phenotypes may require strategies that target neutrophil-driven vascular inflammation rather than generalized immune suppression (150). Conversely, patients with active infection, profound immunosuppression, poor marrow reserve, uncontrolled malignancy requiring intact antitumor immunity, or ongoing immune checkpoint inhibitor therapy should be evaluated cautiously before anti-inflammatory intervention. Thus, anti-inflammatory therapy in CAD–malignancy comorbidity should be framed as endotype-guided immune modulation rather than broad suppression of inflammation.

### 9.2 Antiplatelet and antithrombotic strategies

Platelets and coagulation pathways occupy a central position at the intersection of CAD and malignancy. In CAD, platelet activation and thrombus formation are major drivers of acute coronary syndromes. In cancer, platelets and coagulation factors contribute to cancer-associated thrombosis, tumor-cell survival in circulation, immune evasion, endothelial adhesion, and metastatic dissemination. Therefore, antiplatelet and antithrombotic strategies may have dual cardiovascular and oncologic implications. Palacios-Acedo et al. provided experimental evidence supporting this concept by showing that P2RY12 inhibitors reduce cancer-associated thrombosis and tumor growth in pancreatic cancer (128). This finding is important because P2RY12 inhibition is already a cornerstone of antiplatelet therapy in CAD, suggesting a potential therapeutic overlap between cardiovascular protection and modulation of platelet-driven tumor biology. In selected patients with CAD and malignancy, antiplatelet therapy may therefore influence not only coronary thrombotic risk but also cancer-associated platelet activation.

Coagulation itself may also contribute directly to metastatic progression. Feinauer et al. showed that local blood coagulation drives cancer cell arrest and brain metastasis in a mouse model (130). This finding supports the concept that coagulation is not simply a downstream complication of cancer, but can actively create a permissive vascular niche for tumor-cell arrest and metastatic seeding. Similarly, Li et al. demonstrated that neutrophil extracellular traps participate in cancer-associated thrombosis in gastric cancer, emphasizing the contribution of NET-mediated thromboinflammation (132). However, antithrombotic therapy in cancer patients is complex. The benefits of preventing arterial thrombosis, venous thromboembolism, and platelet-driven tumor interactions must be weighed against bleeding risk, thrombocytopenia, tumor location, invasive procedures, chemotherapy-induced mucosal injury, and drug–drug interactions (151, 152). Importantly, aggressive dual antiplatelet therapy or combined antithrombotic regimens should not be generalized to all patients with CAD–malignancy comorbidity. High-risk oncologic cohorts, including patients with primary central nervous system malignancies, active gastrointestinal mucosal ulceration or bleeding-prone mucosal lesions, and profound chemotherapy-induced thrombocytopenia, should be excluded from intensified antiplatelet or combined antithrombotic strategies, even when they have a high underlying coronary plaque burden, unless there is an overriding cardiovascular indication and multidisciplinary consensus (153, 154). Thus, antiplatelet or anticoagulant strategies in CAD–malignancy comorbidity should be individualized according to cancer type, stage, platelet count, coagulation status, bleeding risk, coronary risk, venous thromboembolism risk, and planned anticancer therapy.

Future studies should determine whether platelet function testing, coagulation biomarkers, NET markers, tumor type, and coronary plaque burden can identify patients who may benefit most from combined cardiovascular and oncologic antithrombotic strategies.

### 9.3 Targeting endothelial activation

Endothelial activation is another important therapeutic target linking CAD and malignancy. In CAD, activated endothelial cells express adhesion molecules, produce inflammatory mediators, recruit leukocytes, and promote platelet adhesion. In cancer, endothelial activation supports tumor angiogenesis, vascular permeability, immune-cell trafficking, tumor-cell adhesion, and cancer-associated thrombosis. Therefore, endothelial dysfunction represents a shared vascular mechanism that may be targeted to reduce both cardiovascular and oncologic complications.

Sano et al. demonstrated that VCAM-1 blockade inhibits pancreatic tumor progression and cancer-associated thrombosis/thromboembolism (129). This study provides direct evidence that endothelial adhesion pathways can regulate both tumor biology and thrombotic risk. VCAM-1 is particularly relevant because it mediates leukocyte adhesion, inflammatory cell recruitment, and tumor–endothelium interaction. In CAD, similar endothelial adhesion pathways contribute to monocyte recruitment into the arterial wall and atherosclerotic plaque formation. Targeting endothelial activation may therefore provide several potential benefits. It could reduce leukocyte recruitment into vascular lesions, limit platelet and neutrophil adhesion, decrease thromboinflammatory signaling, and impair tumor-cell vascular arrest. However, endothelial-targeted approaches must preserve normal vascular repair, immune surveillance, and tissue perfusion. This is especially important in patients receiving antiangiogenic therapies, radiotherapy, or cytotoxic chemotherapy, which can themselves cause endothelial injury.

From a translational perspective, endothelial biomarkers such as VCAM-1, ICAM-1, E-selectin, circulating endothelial cells, endothelial microparticles, and vascular imaging markers may help identify patients with active endothelial inflammation. Combining endothelial profiling with coronary imaging and cancer-associated thrombosis assessment may allow more precise identification of patients at high risk for CAD progression and thromboembolic events.

### 9.4 Immune checkpoint-aware cardiovascular monitoring

The rapid expansion of immune checkpoint inhibitor therapy has created a new need for cardiovascular monitoring strategies that extend beyond traditional cardiotoxicity screening. While myocarditis remains the most recognized ICI-related cardiovascular toxicity, increasing evidence suggests that immune checkpoint modulation may also affect vascular inflammation, plaque immune homeostasis, vasculitis, and potentially accelerated atherosclerosis. Therefore, patients receiving ICIs may require cardiovascular risk stratification beyond myocardial injury markers alone.

Durán et al. demonstrated that human atherosclerotic plaques contain a complex immune checkpoint landscape influenced by cardiometabolic factors (123). This finding suggests that checkpoint pathways may help maintain immune balance within plaques. Blocking these pathways during cancer immunotherapy could potentially disturb local vascular immune regulation, especially in patients with pre-existing CAD, diabetes, obesity, dyslipidemia, smoking history, or systemic inflammation. ICI myocarditis provides a clear example of how checkpoint blockade can trigger cardiovascular immune toxicity. Zhu et al. identified pathogenic immune-cell subsets associated with checkpoint inhibitor-induced myocarditis, while Axelrod et al. demonstrated that α-myosin-specific T cells drive immunotherapy-related myocarditis (146). Although myocarditis and CAD are distinct cardiovascular entities, these studies show that ICIs can break immune tolerance to cardiovascular tissues. This principle may also be relevant to vascular lesions, where autoreactive or antigen-experienced T cells can contribute to plaque inflammation.

The 2022 ESC cardio-oncology guidelines provide a practical framework for translating these mechanistic concerns into clinical workflows for the prevention, diagnosis, monitoring, and treatment of cancer therapy-related cardiovascular toxicity (152, 155). To better align this mechanistic framework with contemporary cardio-oncology guidance, immune checkpoint inhibitor-related cardiovascular monitoring should be embedded within a structured clinical pathway rather than discussed only as a mechanistic risk. According to current cardio-oncology principles, patients should undergo baseline cardiovascular risk assessment before initiation of potentially cardiotoxic cancer therapy, including evaluation of prior CAD, hypertension, diabetes, dyslipidemia, smoking history, obesity, previous cardiovascular events, prior cardiotoxic therapy, and planned combination regimens. Baseline ECG, cardiac troponin, and natriuretic peptide measurement may help establish reference values for subsequent surveillance, while transthoracic echocardiography, including left ventricular ejection fraction and global longitudinal strain when available, should be considered in patients at high or very high cardiovascular risk or those receiving higher-risk regimens. For patients treated with ICIs, serial ECG and cardiac biomarker monitoring during the early treatment cycles may facilitate earlier recognition of myocarditis or other immune-mediated cardiovascular toxicities. In patients with established CAD, high plaque burden, CHIP, diabetes, or systemic inflammation, additional coronary risk assessment, such as coronary artery calcium scoring or coronary CT angiography, may be considered in selected clinical or research contexts. Suspected ICI-related myocarditis or acute coronary events should prompt interruption of ICI therapy, urgent cardiology/cardio-oncology evaluation, cardiac biomarker testing, ECG, echocardiography, and cardiac magnetic resonance imaging when appropriate, followed by multidisciplinary management balancing cardiovascular stabilization with oncologic benefit. Check while labor hematologist.

In summary, therapeutic strategies for CAD–malignancy comorbidity should be mechanism-guided rather than disease-specific. Anti-inflammatory therapy may target CHIP-driven inflammasome activation; antiplatelet and antithrombotic strategies may address platelet–coagulation–NET pathways; endothelial-directed approaches may reduce vascular activation and tumor-associated thrombosis; and immune checkpoint-aware monitoring may identify patients at risk of ICI-related cardiovascular injury. Together, these approaches provide a foundation for precision cardio-oncology, in which cardiovascular protection and cancer control are considered within a unified biological framework. Based on these mechanistic insights, several translational strategies may be considered for patients with CAD–malignancy comorbidity, including anti-inflammatory intervention, antiplatelet or antithrombotic optimization, endothelial-targeted therapy, and immune checkpoint-aware cardiovascular monitoring (**Table 5**).

**Table 5:** Translational strategies and therapeutic interventions in CAD–malignancy comorbidity.

Strategy	Mechanistic target	Patient population/context	Key evidence	PMID
Anti-inflammatory therapy	AIM2/NLRP3 inflammasome, IL-1β	Patients with CHIP, high inflammatory burden, CAD–cancer comorbidity	Colchicine reduces atherosclerosis in TET2-mutant CHIP	Zuriaga MA (39212933), Fidler TP (33731931), Yalcinkaya M (37781816)
Antiplatelet/anticoagulant	P2RY12, platelet activation	CAD patients with cancer or at risk of cancer-associated thrombosis	P2RY12 inhibition reduces thrombosis and tumor growth; NET inhibition reduces thromboinflammation	Palacios-Acedo AL (34589424, 35159000), Li JC (36051331), Feinauer MJ (33270819)
Endothelial-targeted therapy	VCAM-1, ICAM-1	Cancer patients at risk of thrombosis, CAD, or vascular inflammation	VCAM-1 blockade inhibits tumor progression and thrombosis	Sano M (33087490)
Immune checkpoint-aware monitoring	PD-1/PD-L1, CTLA-4, CD40L–CD40	Cancer patients receiving ICI therapy with pre-existing CAD	Plaque immune checkpoint mapping identifies high-risk patients; guides cardiovascular surveillance	Durán JGB (39613875), Zhu H (35762356), Axelrod ML (36385524)
Integrated precision strategy	CHIP, biomarkers, plaque imaging, thrombosis risk	High-risk CAD–cancer comorbid patients	Combines anti-inflammatory, antiplatelet, endothelial, and ICI monitoring interventions	Zuriaga MA (39212933), Palacios-Acedo AL (34589424), Durán JGB (39613875)

## 10 Knowledge gaps and future directions

### 10.1 Causality remains incompletely defined

Although epidemiological studies consistently demonstrate co-occurrence between CAD and malignancy, causality remains incompletely defined (156, 157). Observational associations are vulnerable to multiple confounders, including age, smoking, obesity, diabetes, dyslipidemia, hypertension, systemic inflammation, socioeconomic factors, medication use, cancer stage, and anticancer treatment exposure (158, 159). Surveillance bias may lead to increased cancer detection in patients with CAD because of more frequent healthcare encounters, whereas reverse causality may occur when occult cancer promotes systemic inflammation, hypercoagulability, endothelial dysfunction, or coronary events before cancer diagnosis (160, 161). Therefore, future studies should distinguish shared-risk-factor overlap from true biological mediation and should avoid interpreting CAD–malignancy comorbidity as a uniform or unidirectional causal process.

Longitudinal cohort studies integrating repeated cardiovascular and oncologic assessments are needed to address these questions. Such studies should combine advanced phenotyping, including coronary plaque imaging, systemic inflammatory biomarker profiling, and cardiovascular functional assessment, with detailed cancer staging and treatment exposure data. Mendelian randomization analyses could further clarify genetic and molecular pathways that contribute to the shared risk of CAD and cancer. Sun et al. demonstrated that systemic inflammation modifies the association between CAD severity and cancer risk, providing evidence that biological mediators can strengthen the CAD–malignancy link (11). Similarly, Campolo et al. and Li et al. observed bidirectional associations in large clinical cohorts, supporting the epidemiologic relevance of CAD–malignancy comorbidity. Díez-Díez et al. further demonstrated that clonal hematopoiesis of indeterminate potential (CHIP) is associated with atherosclerosis development, suggesting a mechanistic upstream factor linking hematopoietic clonal evolution to both cancer predisposition and vascular disease (82). Collectively, these studies highlight the need for integrative designs that combine longitudinal clinical observation with molecular and genetic analyses to define causal pathways.

### 10.2 Need for cancer type-specific mechanisms

Emerging evidence indicates that CAD–malignancy comorbidity is not uniform across tumor types. Different cancers exhibit distinct inflammatory, metabolic, thrombotic, and treatment-related profiles, resulting in heterogeneous cardiovascular risk. For example, lung cancer patients often present with chronic pulmonary inflammation, oxidative stress, and endothelial injury, whereas pancreatic and gastric cancers frequently involve hypercoagulable states, platelet activation, and tumor-induced thromboinflammation. Hematologic malignancies and immune therapy-treated tumors may additionally involve clonal hematopoiesis or profound immune remodeling. Zhao et al. demonstrated that the association between CAD severity and lung cancer is strongly influenced by systemic inflammation, highlighting the importance of tumor type and host immune status (12). Palacios-Acedo et al. and Sano et al. provided mechanistic evidence in pancreatic cancer, showing that platelet activation and endothelial adhesion pathways contribute to both cancer progression and cancer-associated thrombosis. Li et al. highlighted the role of neutrophil extracellular traps (NETs) in gastric cancer-associated thrombosis, emphasizing the relevance of tumor-specific thromboinflammatory mechanisms (132). These studies underscore that precision cardio-oncology strategies must consider tumor-specific cardiovascular risk phenotypes. Risk stratification and therapeutic intervention should be informed not only by traditional cardiovascular risk factors, but also by tumor biology, treatment modality, and tumor-specific immunometabolic and thrombotic profiles.

### 10.3 Need for integrated multi-omics and spatial profiling

Mechanistic understanding of CAD–malignancy comorbidity requires high-resolution mapping of cellular, molecular, and spatial interactions. Single-cell RNA sequencing, TCR/BCR sequencing, and spatial transcriptomics can reveal immune-cell composition, clonal expansion, and functional heterogeneity in both atherosclerotic plaques and tumor microenvironments. Integration with proteomics, metabolomics, and imaging mass cytometry can provide additional insight into inflammatory signaling, metabolic remodeling, and thromboinflammatory processes.

Chowdhury et al. characterized clonal T-cell populations within human coronary plaques, revealing cross-reactivity to viral and self-antigens, while Wang et al. demonstrated disruption of tolerance checkpoints at single-cell resolution. Durán et al. mapped the immune checkpoint landscape in human plaques and showed modulation by cardiometabolic factors. Zhu et al. identified pathogenic immune subsets in ICI-induced myocarditis, emphasizing the translational relevance of spatial immune profiling (145). Together, these studies illustrate the potential of integrated multi-omics and spatial approaches to map shared immune and metabolic niches between cardiovascular and oncologic tissues. Such approaches could identify localized inflammatory microenvironments, metabolically primed immune cells, and thromboinflammatory hotspots that simultaneously contribute to plaque instability and tumor progression. This high-resolution mapping is essential for developing targeted interventions that modulate shared pathological pathways without compromising antitumor immunity or cardiovascular homeostasis.

### 10.4 Need for mechanism-guided cardio-oncology trials

Translation of mechanistic insights into clinical practice requires well-designed, mechanism-guided trials. These studies should test whether targeted interventions—such as CHIP-informed anti-inflammatory therapy, antiplatelet or anticoagulant optimization, plaque imaging-guided cardiovascular surveillance, or immune checkpoint-aware monitoring—can improve outcomes in patients with concurrent CAD and cancer. Zuriaga et al. provide proof-of-concept that anti-inflammatory therapy guided by CHIP genotype can reduce atherosclerosis progression (71). Palacios-Acedo et al. demonstrated that P2RY12 inhibition can simultaneously reduce cancer-associated thrombosis and tumor progression, highlighting the dual benefits of antiplatelet therapy (128). Durán et al. underscore the importance of mapping plaque immune checkpoint landscapes to predict cardiovascular risk during immune checkpoint inhibitor therapy. Future trials should stratify patients according to integrated risk profiles that incorporate CHIP status, systemic inflammation, plaque burden, tumor type, therapy exposure, and immune checkpoint activity. This approach will allow evaluation of interventions that simultaneously mitigate cardiovascular events and tumor progression, providing a precision cardio-oncology framework. Additionally, longitudinal follow-up combining clinical outcomes, imaging, and multi-omics readouts will be critical to establish causality, validate biomarkers, and optimize patient selection.

## 11 Conclusion

CAD–malignancy comorbidity is driven by more than shared clinical risk factors. A growing body of clinical, mechanistic, and translational evidence supports a molecular framework in which chronic inflammation, clonal hematopoiesis, inflammasome activation, immunometabolic remodeling, endothelial dysfunction, platelet activation, NET formation, coagulation, and immune checkpoint disruption converge to promote both cancer progression and coronary vascular disease. Understanding these shared pathways has significant clinical implications. It enables a new generation of cardio-oncology strategies that go beyond managing overt therapy-induced cardiotoxicity, focusing instead on the prevention and treatment of underlying molecular mechanisms that simultaneously contribute to cardiovascular and oncologic disease. Integrated approaches—including CHIP-guided anti-inflammatory therapy, antiplatelet/anticoagulant optimization, endothelial-targeted interventions, and immune checkpoint-aware cardiovascular monitoring—represent a precision framework for improving outcomes in patients with coexisting CAD and malignancy. Future research should focus on defining causal pathways, delineating tumor-specific cardiovascular risk, integrating multi-omics and spatial immune profiling, and testing mechanism-guided interventions in prospective clinical trials. Such efforts hold the potential to transform the management of patients at the intersection of cardiovascular and oncologic disease, ultimately improving both survival and quality of life.
